# Chitin Biosynthesis in *Aspergillus* Species

**DOI:** 10.3390/jof9010089

**Published:** 2023-01-06

**Authors:** Veronica S. Brauer, André M. Pessoni, Mateus S. Freitas, Marinaldo P. Cavalcanti-Neto, Laure N. A. Ries, Fausto Almeida

**Affiliations:** 1 Department of Biochemistry and Immunology, Ribeirao Preto Medical School, University of Sao Paulo, Sao Paulo 01000-000, Brazil; 2 Integrated Laboratory of Morphofunctional Sciences, Institute of Biodiversity and Sustainability (NUPEM), Federal University of Rio de Janeiro, Rio de Janeiro 27965-045, Brazil; 3 MRC Centre for Medical Mycology, University of Exeter, Exeter EX4 4QD, UK

**Keywords:** *Aspergillus* spp., chitin, chitin synthase, fungal cell wall, caspofungin paradoxical effect, calcineurin, protein kinase C, cell wall integrity pathway

## Abstract

The fungal cell wall (FCW) is a dynamic structure responsible for the maintenance of cellular homeostasis, and is essential for modulating the interaction of the fungus with its environment. It is composed of proteins, lipids, pigments and polysaccharides, including chitin. Chitin synthesis is catalyzed by chitin synthases (CS), and up to eight CS-encoding genes can be found in *Aspergillus* species. This review discusses in detail the chitin synthesis and regulation in *Aspergillus* species, and how manipulation of chitin synthesis pathways can modulate fungal growth, enzyme production, virulence and susceptibility to antifungal agents. More specifically, the metabolic steps involved in chitin biosynthesis are described with an emphasis on how the initiation of chitin biosynthesis remains unknown. A description of the classification, localization and transport of CS was also made. Chitin biosynthesis is shown to underlie a complex regulatory network, with extensive cross-talks existing between the different signaling pathways. Furthermore, pathways and recently identified regulators of chitin biosynthesis during the caspofungin paradoxical effect (CPE) are described. The effect of a chitin on the mammalian immune system is also discussed. Lastly, interference with chitin biosynthesis may also be beneficial for biotechnological applications. Even after more than 30 years of research, chitin biosynthesis remains a topic of current interest in mycology.

## 1. Introduction

The fungal cell wall (FCW) is a vital and dynamic structure [1], which accommodates changes in morphology, confers plasticity and protects against potentially harmful environmental factors such as osmotic pressure and temperature [1,2]. The FCW is therefore crucial in mediating the interaction of the fungus with the environment and contains a broad number of hydrolytic molecules, which play important roles in specific niche colonization [3]. The FCW composition changes depending on the fungal species. In filamentous fungi, the main CW components are polysaccharides such as glucans and chitin that represent 50–60% and 10–20%, respectively, of the FCW dry weight; as well as glycoproteins, that constitute 20–30% of the FCW dry weight [4]. These components are cross-linked, resulting in a complex network that constitutes the core and structural basis of the FCW [2]. Glucans confer tensile strength to the cell wall, and together with chitins and glycoproteins, they are responsible for the maintenance of the FCW shape [5]. In addition, chitin also confers rigidity to the cell wall, whereas glycoproteins can function in signal transduction, adhesion, absorption of molecules, protection against unfamiliar substances and synthesis and remodeling of the FCW [2,6].

Preceded only by cellulose, chitin is the second most abundant natural polysaccharide in nature [7] and the major component of crustacean shells, insect exoskeletons and FCWs, including the fungal genus *Aspergillus* [7,8]. Chitin was first described in 1811 by Henri Braconnot from raw materials isolated from mushroom species such as *Agaricus volvaceus, A. acris, A. cantarellus, A. piperatus, Hydnum repandum, H. hybridum* and *Boletus viscidus* [8,9]. Chitin is a white and rigid homopolymer, composed of linear β-(1,4)-N-acetylglucosamine (GlcNAc) chains with approximately 6–7% of nitrogen [8]. There are 3 forms of chitin, called α, β and γ, which anneal to each other in different conformations during chitin synthesis. α-chitin, the most abundant form, is when the GlcNAc chains are ordered in an anti-parallel form, whereas parallel stacking of the chains gives rise to β-chitin. In contrast, γ-chitin consists of two or three polysaccharide chains in parallel orientation, whereas the third chain is in the anti-parallel order. γ-chitin is therefore considered a combination of α- and β-chitin [10,11]. The orientation of chitin chains results in an intermolecular hydrogen bond formation, creating fibers that are highly hydrophobic and have a strong tensile force [7,11,12], contributing to the FCW integrity [1].

The interaction between chitin and α-1,3-glucan results in the formation of a highly hydrophobic core in the FCW, which could accommodate pigment molecules, such as melanin [13]. This inner part of the FCW confers rigidity to the structure and is embedded in a hydrated and mobile matrix rich in β-1,3-, β-1,4- and β-1,6-glucans [13]. The structural role of FCW components was also confirmed by Chakraborty et al., 2021 [14], who used different mutant strains lacking chitin, α-1,3-glucan, galactosaminogalactan (GAG) or galactomannan (GM) linked to the β-1,3-glucan-chitin core. A thick cell wall is observed in mutants lacking GAG, GM and α-1,3-glucan. The production of the β-1,3-glucan is increased in the inner part of the FCW in response to the lack of the α-1,3-glucan. In addition, chitin fibril deposition is altered in this mutant lacking the α-1,3-glucan (Δ*ags1* Δ*ags2* Δ*ags3* triple mutant), resulting in a strain with increased rigidity in the FCW. The lack of chitin, as a result of the simultaneous deletion of *csmA*, *csmB*, *chsF* and *chsG* chitin synthase (CS) genes, results in a strain deficient in growth, with the inner domain of the FCW formed basically by α-1,3-glucan and β-1,3-glucan, with no chitin-β-glucan-GAG core formation. The lack of GM results in the absence of proteins and the compensatory increase in GAG and chitin, resulting in a highly hydrophobic and rigid cell wall. The GAG deficient strain does not present so many changes in the inner core of the FCW when compared to the WT strain, but the lack of GAG causes difficulties in water retention, which reflects on the rigidity of the cell wall [14]. This study illustrates the importance of the plasticity and rigidity of the FCW for fungal growth and development.

The genus *Aspergillus* is composed of filamentous saprophytic fungi that are ubiquitous in the soil and air [15]. Several species from this genus are relevant for different industries, including the pharmaceutical and medical sectors [16]. For example, *A. oryzae* and *A. sojae* are used for the production of oriental foods such as soy sauce or sake [17]; whereas *A. niger* is the main producer of citric acid, an organic acid that is used as an acidifier, a food flavoring and a chelating agent [18]. In contrast, *A. terreus* is a prolific producer of statin, a secondary metabolite and hypolipidemic agent, used to treat hypercholesterolemia [17,18]. *A. flavus* and *A. parasiticus* are important food spoilage organisms for a variety of edible crops. Their pathogenicity is characterized by aflatoxin production, a secondary metabolite with hepatotoxicity, teratogenicity and immunotoxicity actions that present a health hazard to humans and cattle [19]. Lastly, *A. fumigatus* is globally recognized as the most important opportunistic human filamentous fungal pathogen. It causes a wide range of diseases in patients with different types of immunodeficiencies, and some of these diseases are accompanied by mortality rates as high as 90% [20].

In this review, we will provide an overview of chitin biosynthesis in *Aspergillus* species while highlighting the need for additional studies to elucidate different aspects (mentioned hereafter) of chitin biosynthesis. We will discuss how chitin is biosynthesized before focusing on the classification and cellular roles of chitin synthases (CS) in different *Aspergillus* species. We will emphasize the importance of CS for fungal morphology, which is intrinsically linked to antifungal drug resistance and interactions with mammalian immune cells. Furthermore, we provide an in-depth discussion of stress- and morphology-related signaling pathways that regulate chitin biosynthesis. Lastly, we will also discuss how CS gene manipulation can be beneficial for biotechnological applications that currently use *Aspergillus* species.

## 2. Chitin Biosynthesis

### 2.1. Metabolic Steps Leading to Chitin Biosynthesis

**Figure 1:**
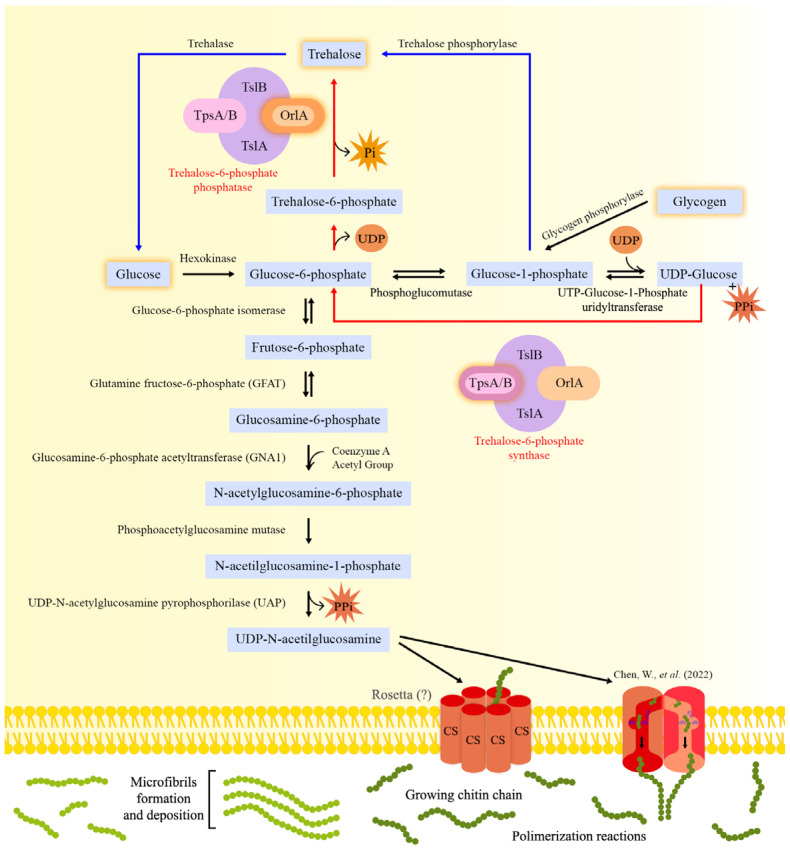
The chitin biosynthesis pathway in *Aspergillus.* Glucose-6-phosphate (G6P) is the precursor for UDP-N-acetylglucosamine (UDP-N-GlcNAc), the main building block of fungal chitin. Glucose is first converted to G6P by hexokinase and then to fructose-6-phosphate (F6P) by G6P isomerase, before an additional 4 enzymatic steps result in the biosynthesis of UDP-N-GlcNAc. The first two steps in UDP-N-GlcNAc biosynthesis are the same as in the glycolytic pathway. The generation of F6P represents the intersection between the glycolytic pathway and chitin synthesis. F6P is converted to glucosamine-6-phosphate by the enzyme glutamine-fructose-6-phosphate (GFAT), marking the beginning of the first and limiting stage of chitin biosynthesis. The enzyme Glucosamine-6-phosphate acetyltransferase (GNA1) catalyzes the addition of an acetyl group, derived from acetyl-CoA, to glucosamine-6-phosphate generating N-acetylglucosamine-6-phosphate, which is then converted into N-acetylglucosamine-1-phosphate by phosphoacetylglucosamine mutase (AGM-1). The next step involves the enzyme UDP-GlcNAc pyrophosphorylase (UAP), which catalyzes the uridylation of GlcNAc-1-phosphate and the formation of UDP-N-GlcNAc, releasing pyrophosphate as a by-product. Chitin synthase (CS), an enzyme found in specialized microdomains of the plasma membrane, uses UDP-N-GlcNAc as a substrate in order to transfer GlcNAc (N-acetylglucosamine) from UDP-GlcNAc to the emerging chitin chains. The growing chitin chains must be translocated across the plasma membrane from the intracellular domain to the outside, a mechanism that remains to be fully elucidated but may involve the participation of molecular complexes termed “Rosettas”. Chen et al., 2022 [64] recently suggested a different mechanism for chitin chain translocation, whereby the CSs present five different conformations: apo, linked to the substrate (UDP-GlcNAc), catalytic, linked to the nascent chitin oligomer, and the product release state. CSs were shown to form a loop that functions as a gate and that prevents the donor substrate from leaving the reaction site without being bound to a growing chitin oligomer. Furthermore, these CSs also direct the new chitin chain to the extracellular space. After reaching the cell wall space, hydrogen bonds form between chitin chains, causing them to be organized into microfibrils. The microfibrils subsequently crystallize in association with other extracellular components, resulting in the formation of the fungal cell wall. In addition, the intracellular carbon storage compounds trehalose and glycogen can also be converted to glucose or G6P, respectively, through a series of enzymatic reactions. The trehalase converts trehalose to glucose, whereas the glycogen is first converted to glucose-1-phosphate (G1P) and then to G6P by the enzymes glycogen phosphorylase and phosphoglucomutase. Furthermore, G1P and G6P derived from glucose or glycogen, respectively, function as precursors for trehalose. G1P is directly converted to trehalose by trehalose phosphorylase, whereas G6P is converted to trehalose by two enzymatic steps involving the large enzymatic complex Trehalose-6-phosphate phosphatase. Red lines: represent the pathway of trehalose production through the transference of glucosyl residues to G6P by the enzyme trehalose-6-phosphate synthase (described in red letters on the figure), releasing trehalose-6-phosphate, that it will be dephosphorylated by trehalose-6-phosphate phosphatase (described in red letters on the figure), forming trehalose. Blue lines: represent the trehalose production through G1P.

Chitin biosynthesis occurs through a process that is conserved in all organisms capable of synthesizing it. In general, the process consists of converting sugars such as glucose (or storage compounds such as glycogen) or the disaccharide trehalose into linear chitin chains [21,22]. These structures are subsequently secreted into the extracellular space, where they aggregate into microfibrils and are organized in the extracellular matrix, allowing the formation of structures such as fungal cell walls [23]. The chitin biosynthesis process is illustrated in Figure 1.

In fungi, the conversion of glycogen into glucose-1-phosphate by the action of the enzyme glycogen phosphorylase represents an important initial step in this process. Glucose-1-phosphate can (1) be converted into glycolysis intermediates or (2) serve as a precursor for trehalose biosynthesis [24]. Glucose-1-phosphate is converted to glucose-6-phosphate by phosphoglucomutase, generating a central glycolysis molecule, which has multiple destinations in cell metabolism [25,26]. In addition, the enzyme Uridine Triphosphate (UTP)-glucose-1-phosphate uridyltransferase catalyzes the production of Uridine Diphosphate (UDP)-glucose and pyrophosphate from glucose-1-phosphate and UDP, while releasing glucosyl residues [27]. Subsequently, the glucosyl residues are transferred to glucose-6-phosphate by trehalose-6-phosphate synthase, resulting in the production of trehalose-6-phosphate, which, when dephosphorylated, results in trehalose production [24]. The trehalose, in turn, can be hydrolyzed by the enzyme trehalase, generating glucose, which will feed into the glycolytic pathway, where it is phosphorylated by hexokinase and converted into glucose-6-phosphate. Subsequently, the glucose-6-phosphate isomerase converts glucose-6-phosphate into fructose-6-phosphate, a molecule that, in association with glutamine, forms the main precursors of UDP-GlcNAc [25] and represents the intersection between glycolysis and chitin biosynthesis.

The first and limiting step in chitin biosynthesis is the formation of glucosamine 6-phosphate from fructose 6-phosphate and glutamine, a reaction catalyzed by the enzyme glutamine-fructose-6-phosphate amidotransferase (GFAT). The importance of GFAT in the synthesis of chitin has been shown by GFAT-encoding gene (*gfa1*) expression analyses in different microorganisms and higher eukaryotes. In *S. cerevisiae*, genetically or chemically induced cell wall stress strongly induced the expression of *gfa1* [28]. Similarly, calcofluor white (CFW) and caspofungin (CP)-induced CW stress increased *gfaA* mRNA levels in *A. niger*, *Penicillium chrysogenum* and *Fusarium oxysporum*. An increase in chitin levels in the *A. niger* CW after incubation with CFW was also observed. These results support the induction of chitin biosynthesis as a general response mechanism to cell wall stress [29].

The second step includes the formation of N-acetylglucosamine (GlcNAc)-6-phosphate, through the donation of the acetyl group derived from coenzyme-A to Glycosamine-6-phosphate, by the action of the enzyme glucosamine-6-phosphate acetyltransferase (GNA1). Acetylation is characteristic of N-acetylglucosamine (GlcNAc) polymers, and it is one of the main differences in the structure and physicochemical properties when comparing chitin and cellulose molecules [30]. Computational studies have revealed the role of acetylation in the formation of hydrogen bonds, which is important for the crystalline structure of chitin, which confers a strong resistance to physical damage [31]. In vitro studies with *A. fumigatus Δgna1* revealed that this strain was unable to grow in culture in the absence of exogenously added GlcNAc, suggesting that GNA1 is crucial for *A. fumigatus* survival, representing a potential target for the development of antifungal agents [32].

Subsequently, phosphate is transferred from C6 to C1 by the action of the enzyme phosphoacetylglucosamine mutase (termed Agm1 in *Aspergillus* spp.), resulting in the formation of N-acetylglucosamine (GlcNAc)-1-phosphate [25,33]. Agm1 was shown to be an essential enzyme in *A. fumigatus* through the construction of a conditional mutant [34]. In this strain, the *agm1* open reading frame (ORF) was placed under the control of the *alcA* (alcohol dehydrogenase) promoter. In the presence of glucose (the repressive condition), *agm1* is repressed, whereas the removal of glucose and the addition of other carbon sources (the inductive condition), including ethanol, results in *agm1* transcription. The lack of *agm1* expression resulted in a thinner cell wall in both conidia and hyphae, with the conidia being unable to retain the melanin layer. Indeed, analysis of the cell wall in the *agm1* conditional mutants showed increased levels of α-glucan and β-glucan and lower levels of glycoproteins and chitin when compared to the wild-type strain. Interestingly, crystallography analyses revealed several amino acid changes near the substrate binding site in *A. fumigatus* AGM1 when compared to the human homologue, making this enzyme a potential target for the design of fungus-specific selective inhibitors [34].

The next step in chitin biosynthesis is catalyzed by UDP-GlcNAc pyrophosphorylase (UAP), a nucleotidyltransferase characteristic of the metabolism of aminosugars, which catalyzes the formation of UDP-GlcNAc through the uridylation of GlcNAc-1-phosphate. During this reaction, pyrophosphate is released, and UDP-N-acetylglucosamine, a substrate for CS, is generated [35,36]. In *A. fumigatus*, the *uap1* gene is essential, and a conditional mutant was constructed for additional characterization [34]. Upon *uap1* gene repression, a considerable reduction in cell growth, accompanied by structural changes in conidia and hyphae, including cell wall thinning and reduction of the content of α-glucan, chitin and GlcNAc in the mycelial cell wall was observed when compared to the WT strain. In addition, the presence of cell wall-interfering compounds such as Congo red, CFW, SDS and hygromycin B increased sensitivity to these reagents when *uap1* expression was repressed, suggesting that the reduction of *uap1* expression results in impaired cell wall integrity. Indeed, the mycelial α-glucan and chitin levels, as well as, GlcNAc were reduced by 36%, 11% and 23%, respectively, in the conditional mutant strain when compared to the WT strain, suggesting that Uap1 is crucial for the cell wall structure and integrity [34].

All aforementioned enzymatic steps occur in the cytoplasm [25], whereas all subsequent steps involving chitin polymerization occur in specialized microdomains of the membrane on the growing buds of yeast cells [37] and at hyphal tips [38]. The generated UDP-N-acetylglucosamine (GlcNAc) molecules serve as a substrate for CS, which will catalyze the transfer of the sugar moiety of UDP-GlcNAc and promote the formation of growing chitin chains. CS is considered the only enzyme specifically committed to chitin biosynthesis and, for this reason, has been described as the key enzyme in this process [25,39]. CSs are integral membrane-bound glycosyltransferases, that mediate the transfer of GlcNAc from the nucleotide uridine diphosphate (UDP)-GlcNAc to a chitin chain in linear expansion, subsequently releasing UDP as a by-product, which requires a divalent ion for its activity [2,25,40,41,42]. CSs are located in the Golgi complex and plasmatic membranes, as well as in chitosomes, which are intracellular vesicles responsible for transporting these enzymes from the endoplasmic reticulum to the cell surface [41,43,44,45]. They are crucial for fungal growth and developmental processes, being intrinsically associated with the cell wall, mechanisms of morphogenesis, growth and hyphal differentiation and conidia formation [41,46].

### 2.2. Initiation of Chitin Biosynthesis: An Unknown Mechanism

The molecular mechanisms involved in chitin synthesis initiation and deposition still require further investigation due to the lack of structural data as well as knowledge about post-catalytic events [47]. The biochemistry and reaction mechanisms that underlie chitin biosynthesis were mainly obtained from CS and related glycosyltransferase studies [25]. The currently proposed CS enzymatic mechanism involves the transfer of GlcNAc from the donor sugar nucleotide UDP-GlcNAc to the non-reducing end of an extended glycan chain. The process takes place through an SN2-like displacement reaction. The C4 hydroxyl group at the non-reducing end of the acceptor glycan attacks the anomeric C1 of the sugar bound to the donor UDP, releasing UDP. The polymerization involves a reaction mechanism where the nucleophilic attack by the acceptor hydroxyl group results in an inversion of the anomeric carbon of the donor substrate [48,49,50].

How polymerization starts during chitin synthesis has not been fully elucidated. Hypotheses suggesting the requirement for soluble or covalently bonded primers for polymerization initiation remain unproven [51]. Chitin polymerization may be mediated by CS itself or by an associated protein when using glycogen as a primer [45,52]. Possible initiation mechanisms involve the generation of a UDP-linked disaccharide, resulting from a reaction between two UDP-linked saccharides, or monosaccharide transfer from a UDP sugar to some low molecular weight initiator molecule. However, given that the UDP-linked disaccharide represents an initiation product characteristic of a reducing-end extension mechanism, this hypothesis conflicts with the framework-based model for elongation of the non-reducing end chain [53]. Gyore et al., 2014 used different strains of *Saccharomyces cerevisiae*, which expressed a single chitin synthase, Chs2, to demonstrate that the formation of chitin oligosaccharide (CO) chains by these enzymes can be stimulated by the addition of GlcNAc and 2-acylamido analogues, and that the latter molecules act as acceptors for the transfer of GlcNAc from UDP-GlcNAc. In addition, the study revealed that 2-acylamido analogues can stimulate the synthesis of insoluble chitin. The authors conclude that CS uses GlcNAc analogues as primers and CO formation occurs by transferring GlcNAc units at a time. The study strongly suggests that GlcNAc itself can initiate the formation of CO and chitin in vitro [54]. Similar studies have not yet been conducted in *Aspergillus* spp.

Once the cell wall space has been reached, hydrogen bonds occur between chitin chains, and microfibril structures are formed, allowing subsequent crystallization [2] that associates with other extracellular components, resulting in the formation of the FCW [55]. Before the chitin chains are organized into microfibrils and deposited on the cell surface, they must first be translocated across the plasma membrane [56]. The biochemical and ultrastructural data from the fungal systems revealed that the catalytic machinery involved in chitin chain translocation is composed of an assembly of tightly packed membrane-bound polymerizing enzymes with cytoplasmic exposure of the catalytic site [57,58] and, as a consequence, the translocation of chains of chitin can occur from the intracellular domain outwards. However, the mechanism of translocation across plasma membranes has not been fully elucidated, and some hypothetical models have been proposed [55,59] (Figure 1).

One of these hypothetical models is based on the structural organization and topographical location of cellulose synthase, an enzyme analogous to CS. Transmission microscopy analysis of freeze-fractured membranes revealed that cellulose synthase has a hexagonal configuration composed of six particles, called “Rosetta” structures [60,61,62]. These structures show transmembrane segments constituting the cellulose synthesis machinery. In addition, it is observed that these protein complexes are organized in the shape of a pore through which, supposedly, the cellulose chains are translocated. Data obtained from the analysis of the enzyme hyaluronan synthase, a glycosyltransferase that catalyzes the transfer of repeated disaccharide units of hyaluronic acid and GlcNAc, and subsequent formation of the biopolymer hyaluronan, also suggest that the transmembrane domains present in these structures present a pore-like arrangement [63]. These studies lend theoretical support and corroborate the hypothesis of the formation of a pore-shaped complex in studies that show the presence of CS transmembrane domains integrated into the cell membrane of different organisms; although the actual structure of the complex catalyzing chitin chain translocation remains to be defined [55].

Recently, structural and functional data based on Chs1 from *Phytophthora soybean*, a pathogenic oomycete that causes root and stem rot in soybean, were found to contribute to the mechanistic understanding of chitin biosynthesis at the atomic level [64]. The analysis of *Ps*Chs1 by cryo-electron microscopy (cryo-EM) identified five structures that reflected different states of the enzyme: apo, linked to the substrate (UDP-GlcNAc), catalytic state, linked to the nascent chitin oligomer, and the product release state. In the apo state, the channel that connects the extracellular side of the membrane with the intracellular reaction chamber of the CS is blocked by a loop that controls chitin access to the reaction chamber. Chitin synthesis initiates when UDP-GlcNAc binds to the substrate-binding site, followed by its hydrolysis, releasing GlcNAc, or when the substrate is exogenously added to the system. This first GlcNAc molecule remains trapped inside the chitin-translocating channel, whereas the second added GlcNAc molecule is at the entrance and extends outside of the channel. As a result, the “loop gate” that blocked the chitin-translocation channel opens. In addition, these changes in loop conformation also prevent the donor substrate from leaving before it can bind to a growing chitin oligomer, and direct the new chitin chain towards the extracellular side of the plasma membrane. Finally, after many reactions, the enzyme changes its conformation to the “post-released state”, where the chitin chain is released from the CS. Subsequently, the loop gate blocks the chitin-translocated channel again, until a new substrate comes, re-starting the cycle of chitin chain biosynthesis [64].

An alternative hypothesis for chitin chain export involves the participation of microvesicular organelles called chitosomes. They are vesicles with a diameter of approximately 40–70 nm, supposedly originating from the endoplasmic reticulum and Golgi complex. It is suggested that chitosomes contain multi-units of the CS polymerizing enzyme [44] and for this reason, they would have an important role in the processes of trafficking individual or packaged CS units [55] as well as in the synthesis of chitin polymers [65,66]. However, the idea that the chitosomal compartment could function as a vehicle for CS clusters and assist in the translocation of chitin chains does not represent a plausible strategy, since the fusion of the chitosomal vesicle with plasma membranes does not provide a solution as to how chitin chains are translocated across the plasma membrane. Among the limitations of the hypothesis is the size of the vesicles, which are too small to store chitin chains. In addition, the entry of substrate molecules into the vesicles would require the existence of specific transport mechanisms in the membranes of chitosomes [55,67].

## 3. Chitin Synthases in *Aspergillus* Species

Reverse genetic assays, including CS gene disruptions, deletions, complementation and interference RNA (RNAi) studies [68,69,70,71,72,73], showed that different CS genes are expressed during different fungal cell cycle stages, at different sites of fungal structures during vegetative growth, and can be induced by different environmental conditions, indicating distinct functionality between them [74,75].

**Figure 2:**
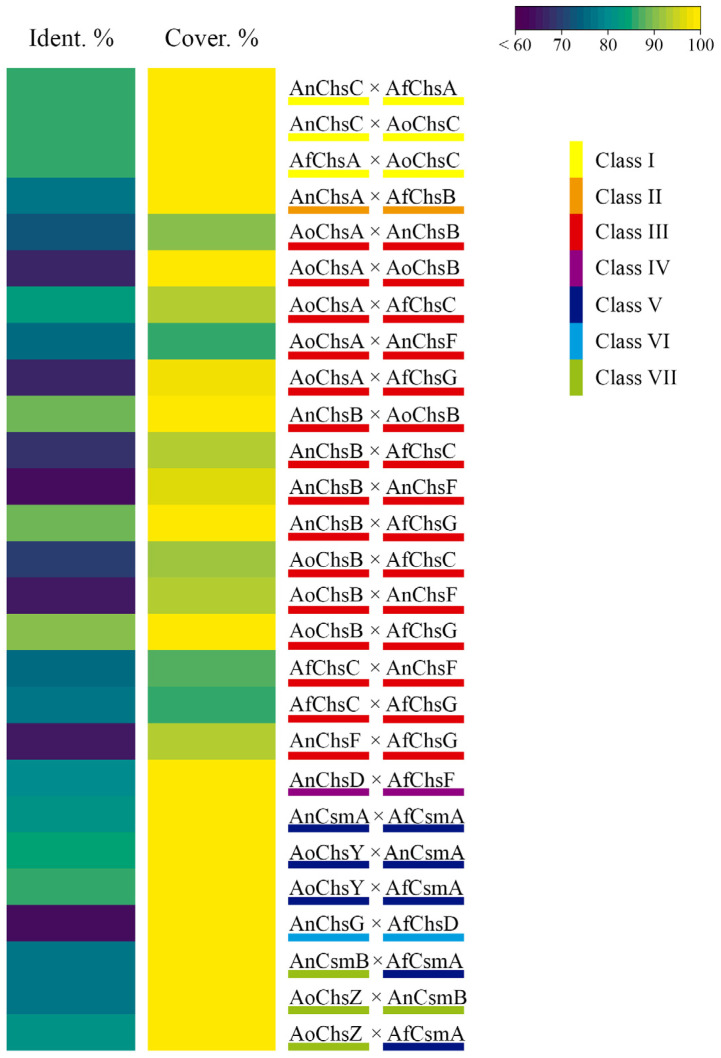
Chitin synthases (CS) of different classes are highly conserved amongst *Aspergillus* species. A heat map depicting the percentage (%) of identity and coverage between protein sequences of CS from the same classes of different *Aspergillus* species. The protein sequences were retrieved from the FungiDB database before pairs were submitted to BlastP for alignment analysis (BLAST Tool NCBI). An, *Aspergillus nidulans*; Af, *Aspergillus fumigatus*; Ao, *Aspergillus oryzae*. On the right, different coloured bars underlining the *Aspergillus* CSs are compared with each other, indicating the class of CS. On the left, heat maps depicting the percentage of identity and coverage between two CS protein sequences from different *Aspergillus* species.

Figure 2 compares the protein sequences of *Aspergillus* spp. orthologous CS-encoding genes. Orthologs perform similar functions between species, although the reverse is not always true [76], and we will see in the next topic that similar functions can be attributed to CS gene orthologs.

### 3.1. Classification of Chitin Synthases (CS)-Encoding Genes

In *S. cerevisae*, the three CS genes *chs1, chs2 and chs3* are well described [41], where they encode enzymes with distinct functions during cell wall expansion, budding and septum formation [77]. CS genes from *S. cerevisiae* are expressed in a regulated manner during the different phases of the cell cycle [78], with increased expression of *chs1* in the M/G1 phase, *chs2* in the M phase and *chs3* right before cell division [79]. During early growth and cell budding, chitin synthesis is required at the bud tip; after the budding process and during isotropic growth, chitin is distributed all across the nascent fungal cell [80]. Following nuclear cell division, chitin biosynthesis is directed to the mother-bud neck, at the site of the bud scars [81]. Yeast CS gene expression is required on the bud site in order to restart the yeast growth/division cycle [82]. The situation is more complex in filamentous fungi, where chitin synthesis is governed by a higher number of CSs, since chitin content in filamentous fungi is significantly higher (10–20%) when compared to *S. cerevisiae* (1–2%) and *Candida albicans* (2–5%) [4,80]. Filamentous fungi undergo a more complex vegetative growth and exhibit elaborate sexual cell cycles, requiring a different demand for chitin synthesis, which is reflected in a differential expression of CS genes at distinct fungal sites, such as the apical tip or during septum formation [25,79,83]. 

CS can be classified into divisions and classes (Table 1) based on their protein sequence, phylogenetic position and similarities in catalytic domains [84,85]. Several studies have been published about CS classification, and as a consequence, the nomenclature became heterogenous, with differences in the number of divisions and classes [3,41,86,87,88,89]. In an attempt to standardize and update CS classification, Gonçalves and colleagues (2016) performed a multi-species comparative analysis (eukaryotes, bacteria and viruses) and showed that fungal CSs are grouped into two divisions (1 and 2), supporting the study carried out by Niño-Vega et al., 2004 [85,88]. CSs from division 1, which include classes I, II and III, have a PF01644 domain, a type 1 CS catalytic domain (CS1) and a PF08407 CS N-terminal domain (CS1N). The members of division 2, which include classes IV, V and VII, comprise enzymes with a catalytic domain preceded by the PF00173 domain, which is a cytochrome b5-like binding domain with a predicted function of binding to lipid ligands [83,85]. Furthermore, they also possess a type 2 CS domain 2 (PF03142) and a DEK C-terminal domain (PF08766) with unknown functions. Classes V and VII of CSs further have an N-terminal myosin-motor such as the PF00063 domain [90], which is associated with the intracellular trafficking of the CS and the site of chitin secretion [91]. CSs of class VI are the only enzymes to have a PF03142 domain [84,90], and they are clustered separately from the other CS classes. Ninõ-Vega et al., 2004 classified the class VI CS into Division 2, but Gonçalves et al., 2016 suggested that a recombination event between two family 2 glycosyltransferases might be the origin of this class of CS. Class VI CS protein sequences have a duplication of the QXXEY motif, a C-terminal region similar to other CSs, and an N-terminal region similar to the one found in hyaluronan synthase [85]. Alternatively, the ancestor of this class of CS underwent accelerated evolution, a process that blurred the phylogenetic signal [85]. Due to the two aforementioned hypotheses and the fact that no CS activity has been shown for this class of CS, these CSs are termed “recCS” (recombined CS) [85]. 

The number of CS genes varies depending on the fungal species, with eight CS genes being present in the genome of *A. nidulans, A. fumigatus* and *A. niger*, and five CS genes encoded in the genome of *A. oryzae*. Table 1 lists each gene from these species, together with gene IDs according to the FungiDB database, whereas division and classes are according to Niño-Vega et al., 2004. We subsequently adopted this nomenclature in this review.

### 3.2. Aspergillus Mutants of CS Genes from Division 1: Morphological Features

In *A. nidulans*, deletion of the class I CS *AnchsC* resulted in a strain with no defects in growth, fungal morphology and conidiation [69]. This is similar to the *A. fumigatus* class I *chsA* (Afu2g01870) deletion strain [103]. In *A. nidulans* and *A. fumigatus*, deletion of the class II CSs Δ*AnchsA* and Δ*AfchsB* did not result in any growth defects, although the Δ*AnchsA* strain has defects in conidiation and a significantly decreased hyphal chitin content [68,103,104]. The double ∆*AnchsA* ∆*AnchsC* mutant presented growth and morphological defects when compared to the respective single deletion strains [105]. In addition, this strain was highly sensitive to SDS (sodium dodecyl sulphate), chitin-binding dyes and chitin synthase inhibitors [105]. Furthermore, the ∆*AnchsA* ∆*AnchsC* strain presented a heterogeneous and dense lateral cell wall, electron-transparent regions situated immediately outside the membrane, denser septa and large septal spores when compared to the WT and single deletion strains, suggesting abnormalities in the hyphal cell wall [106]. Together, these results suggest that the *An*ChsA and *An*ChsC CSs have overlapping functions during the conidiation process [93] and cell wall development as well as having redundant functions in septum formation and development [105,106].

Deletion of the class III CS *An*ChsB (AN2523) resulted in a strain unable to form colonies and conidia and to sustain hyphal growth in the presence of an osmotic stabilizer [68,107]. A similar phenotype was also observed when the promoter region of *AnchsB* was substituted by the *alcA* promoter (*alcA(p)*::*AnchsB*). Under repressing conditions, the *alcA(p)*::*AnchsB* strain presented growth defects, hyphal morphological abnormalities and abnormal asexual development [108], suggesting that this CS is important for all developmental stages of the fungal life cycle. A similar functional activity of *An*chsB orthologs during hyphal development, growth and conidiation was also observed in *A. fumigatus* [Afu3g14420 (ChsG] and *A. oryzae* [AO090701000589 (ChsB)]. *Af*ChsG and *Ao*ChsB are approximately 89% identical to *An*ChsB at the protein level (Figure 2). Deletion of the respective genes resulted in strains exhibiting compact colonies, hyperbranching hyphae, and aberrant conidia formation [72,97,103,109]. In addition, ∆*AfchsG* conidia did not mature and presented a disorganized cell wall with a poorly adherent layer of melanin and showed decreased levels in GlcNAc. Moreover, significantly decreased CS activity was observed for this deletion strain when compared to the WT strain, suggesting that *Af*ChsG is the major contributor of CS activity in *A. fumigatus* [97,103,109]. Deletion of *AfchsC* (Afu5g00760), encoding an additional class III CS in *A. fumigatus* and with a 76.94% similarity to *AfchsG*, did not cause obvious morphological and growth abnormalities, although a reduction of the GlcNAc content in the conidia cell wall was observed [97,103]. The simultaneous deletion of *AfchsG* and *AfchsC* led to decreased colony and conidiophore length, increased hyphal density, and decreased CS activity when compared to the WT strain [97], suggesting that, *Af*ChsG is also important for *A. fumigatus* growth and conidiation.

The genetic relationship between *AnchsA* and *AnchsB*, and *AnchsC* and *AnchsB*, using the *alcA(p)*::*AnchsB* strain [110] was also analyzed with regards to hyphal morphology, growth rate and chitin content [111]. The *alcA(p)*::*AnchsB* ∆*AnchsA* strain presented similar growth when compared to the *alcA(p)*::*AnchsB* strain, but the colony color was dark brown and fewer aerial hyphae were produced. In addition, the *alcA(p)*::*AnchsB* ∆*AnchsA* strain presented increased branching when compared to the parental strains. These results suggest that both *A. nidulans* ChsB (class III) and ChsA (class II) have distinct roles and do not have redundant functions during growth and development. The *alcA(p)*::*AnchsB* ∆*AnchsC* strain also presented similar growth when compared to the *alcA(p)*::*AnchsB* strain, although the hyphal mass was decreased in the periphery of the colony. In addition, the hyphae showed a disorganized pattern, and the sparse growth at the periphery of the colony was abolished by the addition of CFW and Congo red, resulting in a decrease in colony diameter when compared to the parental strains. These studies suggest that the *A. nidulans* CSs ChsB (Class III) and ChsC (class I) have distinct and redundant (deletion of *chsC* alone does not affect growth and morphology) functions during fungal growth and hyphal morphogenesis. Supporting these results is the observation that chitin content was significantly increased in both the *alcA(p)*::*AnchsB ∆AnchsA* and *alcA(p)*::*AnchsB* ∆*AnchsC* strains, suggesting that the simultaneous absence of two CSs resulted in a different cell wall composition. Subsequently, when *AnchsA* was expressed ectopically under the control of the *AnchsB* promoter, before growth rates, colony and hyphal morphologies were determined and found to be similar to the parental strain, suggesting that *An*ChsA and *An*ChsB have non-redundant and non-overlapping functions [111].

**Figure 3:**
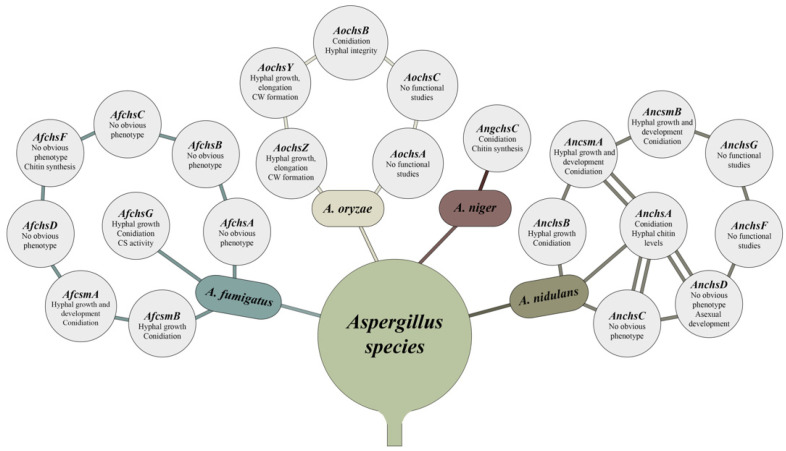
An overview of the functions of chitin synthases (CS) in different *Aspergillus* species. The functional description is based on studies using the respective gene deletion strain. The diagram also depicts CS whose gene deletion has not been carried out and, thus, where no functional studies were performed. Our understanding of the role of CS in *Aspergillus* species is mainly derived from *A. nidulans* and *A. fumigatus*. Overlapping functions between *AnchsA* and *AnchsC* in hyphal growth and conidiation, between *AnchsA* and *AnchsD* in conidiation and between *AnchsA* and *AncsmA* in hyphal integrity under low osmotic conditions in *A. nidulans* are depicted with a double line.

With the aim to clarify the function of each *A. fumigatus* CS from division 1, Muszkieta and colleagues (2014) constructed single and multiple CS deletion strains. The ∆*AfchsG* (Class III) and the ∆*AfchsA* (Class I) ∆*AfchsC* (Class III) ∆*AfchsB* (Class II) ∆*AfchsG* (Class III) strains had reduced growth when compared to the WT strain, and together with the ∆*AfchsA* ∆*AfchsC* and ∆*AfchsA* ∆*AfchsC* ∆*AfchsB* strains also presented increased hyphal branching. Furthermore, the ∆*AfchsA* ∆*AfchsC* ∆*AfchsB* ∆*AfchsG* strain produced abnormal conidiophores and significantly fewer conidia, even in the presence of KCl, when compared to the single deletion and WT strains. Conidia from the ∆*AfchsG* and ∆*AfchsA* ∆*AfchsC* ∆*AfchsB* ∆*AfchsG* strains were swollen, with thin, disorganized cell walls and a melanin layer that was loosely attached to the adjacent cell wall. In addition, the ∆*AfchsG* and ∆*AfchsA* ∆*AfchsC* ∆*AfchsB* ∆*AfchsG* strains presented significantly reduced CS activity when compared to the WT strain. No significant reduction in GlcNac was observed for all mutant strains, but a reorganization of the mycelial cell wall is predicted to occur, as levels of β-1,3-glucan decreased and α-1,3-glucan concentrations increased in all single deletion strains and the ∆*AfchsA* ∆*AfchsC* ∆*AfchsB* ∆*AfchsG* strains. In contrast, GlcNac concentrations were significantly reduced in the cell wall of conidia in all single deletion strains and in the ∆*AfchsA* ∆*AfchsC* ∆*AfchsB* ∆*AfchsG* strain. Together, these results suggest that (i) the *Af*ChsG CS accounts for a large proportion of total CS activity and chitin biosynthesis and (ii) that the other division 1 CSs have less important and/or redundant roles in chitin biosynthesis during asexual development in *A. fumigatus* [103]. This is in contrast with *A. nidulans*, where CS have both distinct and redundant roles; although it is difficult to draw this comparison as a strain deleted for all division 1 CSs has not been generated in *A. nidulans*. Figure 3 summarizes CS gene functions during the fungal development.

### 3.3. Aspergillus Mutants of CS Genes from Division 2: Morphological Features

Deletion of class IV CS did not result in obvious phenotypes. The ∆*AnchsD* (AN1555) strain presented no defect in the production of conidia, sexual structures such as ascospores and cleistothecia, cell growth and morphology [43,92]. The ∆*AnchsD* strain had a 35% reduction in chitin content when compared to the WT strain, irrespective of whether an osmotic stabilizer was added or not [43]. A similar phenotype was also observed for the *A. fumigatus AfchsF* (Afu8g05630) deletion strain, coding for a CS with 79.62% of identity to *An*ChsD. No defects in conidiation, growth and hyphal morphology were observed, although a reduction in mycelial chitin content was observed for this mutant strain [103]. These results suggest that *Aspergillus* can accommodate a certain loss of cell wall chitin or perhaps compensate for the loss of chitin by increasing the concentration of other cell wall polysaccharides as is the case during the caspofungin paradoxical effect (CPE, see below).

The simultaneous deletion of genes encoding *An*ChsD and the class I CS *An*ChsA, resulted in a strain with reduced conidia formation, suggesting that *An*ChsA and *An*ChsD have redundant functions during conidia formation in *A. nidulans* [68,92,104]. Furthermore, fewer conidiophores were also observed under repressing conditions when the promoter region of *AnchsB* was replaced with the *alcA* promoter in the ∆*AnchsD* background strain [110]. Reduced growth rates and abnormal hyphae were also observed, suggesting that the lack of *An*ChsB increases the functional importance of *An*ChsD during growth (mainly in high osmolarity conditions) and during asexual reproduction [110].

#### 3.3.1. Chitin Synthases with Myosin-Motor Like (MMD) and Chitin Synthase Domains (CSD)

CSs from class V (*An*CsmA, *Af*CsmA, *Ao*ChsY) and class VII (*An*CsmB, *Af*CsmB, *Ao*ChsY) contain an N-terminal myosin-motor like domain (MMD), in addition to the C-terminal chitin synthase domain (CSD). 

The role of the *An*CsmA MMD in cell wall assembly and integrity was investigated through the construction of several different mutants, including a deletion mutant, the N630 strain (deletion of the 1220 C-terminal amino acids, expressing only the 630 N-terminal amino acids); the CH5 and CH9 strains, where the CS domain was put under the regulatory control of the *alcA* promoter; the CS3 and CS5 strains, where the entire protein was under the regulatory control of the *alcA* promoter; as well as the ∆*AnchsD* (Class IV) ∆*AncsmA* (DM-3) strain [112]. The ∆*AncsmA* strain did not form cleistothecia during self-mating, aerial mycelia, conidiophores and conidia, independent of the presence of an osmotic stabilizer [43]. In addition, the ∆*AncsmA* strain presented aberrant growth, abnormal hyphal morphology with balloon-like structures and intrahyphal hyphae features, and abnormal septum formation. The hyphal balloon shape and intrahyphal hyphae were also seen when the CH5 strain was cultivated in *AncsmA* inducible conditions, suggesting that the *An*CsmA CS is important for the maintenance of the hyphal cell wall and polarized cell wall synthesis, but that the MMD is dispensable for the formation of normal hyphae. No phenotypical differences were observed between the ∆*AncsmA* and ∆*AnchsD* ∆*AncsmA* strains, with the latter also presenting hyphae with morphological abnormalities; thus, suggesting an overlapping function between these CSs. In agreement with the function of *An*CsmA in cell wall integrity, Yamada et al., 2005 showed that *An*CsmA and *An*ChsA have overlapping functions in maintaining hyphal cell wall integrity mainly in the presence of low osmotic conditions. In addition, the growth of the triple deletion mutant ∆*AnchsA* ∆*AnchsC* ∆*AncsmA* was extremely slow, accompanied by abnormal hyphal morphology, abnormal chitin deposition and irregular septa distribution in low osmotic conditions. Furthermore, an increase in mRNA levels of *AncsmA* in high osmotic conditions was observed for the ∆*AnchsA* ∆*AnchsC* strain when compared to the WT, ∆*AnchsA* and ∆*AnchsC* strains. Altogether, these data suggest that *An*CsmA is important for polarized growth and also may have a role in cell wall repair and maintenance, as mRNA levels of *csmA* are expressed in the presence of osmotic stress [113].

With 82.13% of identity, the *A. fumigatus Af*CsmA (Afu2g13440) CS is orthologous to *An*CsmA. Deletion of *AfcsmA* resulted in a strain with reduced growth when compared to the parental strain [103]. In addition, the ∆*AfcsmA* strain exhibited 70–80% of intrahyphal growth, a decreased sporulation process, conidia with permeable cell walls, less chitin content and a reduced number of conidiophores with altered morphology. Medium supplemented with osmotic stabilizer agents (such as KCl or sucrose), partially restored conidiation of this strain [71]. Alsteens and colleagues (2013) subsequently analyzed architecture, hydrophobicity and polysaccharide concentrations of cell walls from conidia of this mutant strain. In contrast to *A. fumigatus* WT conidia, conidia from the Δ*AfcsmA* strain had low amounts of homogenous rodlets and presented an amorphous and granular surface, with tip-induced alterations, suggesting that Δ*AfcsmA* conidia are more fragile [108].

Some of the growth and developmental abnormalities observed for the Δ*AfcsmA* strain were similar to the Δ*AfchsG* strain, deleted for a class III CS-encoding gene. The double deletion of Δ*AfcsmA* and Δ*AfchsG* strains presented decreased radial growth when compared to the WT and Δ*AfcsmA* strains but grew similarly than with the Δ*AfchsG* strain. In addition, colonies from the double deletion strain were more compact, with increased hyphal branching and severely decreased sporulation in the absence of the osmotic stabilizers KCl or sorbitol. Conidia of the Δ*AfcsmA* and Δ*AfchsG* strains were spherical and pear-shaped, respectively, when compared to conidia of the WT strain, and germination rates were significantly increased. Furthermore, chitin synthase activity decreased, and chitin content was also severely reduced in the double deletion strain when compared to the parental strain. The reduction in chitin resulted in an increase in other cell wall polysaccharides, such as α-glucan. It is possible that the genetic reduction in chitin destabilized the cell wall glucan content and structures, highlighting the importance of CsmA and ChsG for *A. fumigatus* hyphal growth, development and sporulation [109].

*A. oryzae* also contains a CS-encoding gene, *chsY* (AO090026000323), that has high similarity at the protein level with *An*CsmA (83.97%) and with *Af*CsmA (86.23%). This gene codes for class V CS, and is predicted to be important for hyphal growth, elongation and cell formation. Expression of *AochsY* occurred mainly in liquid cultures with complete medium, whereas transcription of this gene was reduced in solid and minimal medium [100]. To date, no studies investigating the role of *AochsY* in fungal growth and development have been carried out.

The CS from class VII, are encoded by the genes *AncsmB* (AN6317) in *A. nidulans, AfcsmB* (Afu2g13430) in *A. fumigatus*, and *AochsZ* (AO09002600321) in *A. oryzae*. The CSD is highly conserved between the species, when compared to the MMD. The CSD from *An*CsmB has 55% similarity with the class V CSD from *An*CsmA [114]. Similarly, in *A. fumigatus*, the *Af*CsmB CSD has 60% similarity with the class V *Af*CsmA CSD [71]. In contrast, MMD significantly differs between class VII CS, with MMD from *An*CsmB and *An*CsmA having 21% similarity with each other [114], and 11% similarity between MMD from *Af*CsmA and *Af*CsmB [71]. The MMD domain from class VII CS is smaller than the MMD from class V CS, and the ATP-binding motifs are not conserved [100,114]; hence the classification of *An*CsmB, *Af*CsmB and *Ao*ChsZ as class VII of CS. 

Intrigued by the similarities and differences of the MMD and CSD from classes V and VII CS, Tsuizaki and colleagues, 2013, constructed strains that produce chimeric proteins in order to further investigate the function of these domains. They fused the MMD from *An*CsmA to the CSD from *An*CsmB in a strain termed MACB, and fused the MMD from *An*CsmB to the CSD from *An*CsmA in a strain named MBCA. Constructions were placed under the control of the *An*CsmA or *An*CsmB promoter regions in strains that were deleted for the open reading frame (ORF) of either *An*CsmA (strains were termed MACB∆A1 and MBCA∆A1) or *An*CsmB (strains were named MACB∆B1 and MBCA∆B1). The MACB∆A1 strain had increased chitin content, balloon-shaped hyphae with increased lysis at subapical regions, and presented growth rates that were significantly reduced when compared to the WT. In contrast, no differences in growth rates between the MBCA∆A1 and WT strains were observed, although the mutant strain presented some hyphal abnormalities and reduced conidiation. These results suggest that the *An*CsmB CSD cannot substitute for the *An*CsmA CSD, but that the MMDs have similar functions in both CSs. The MACB∆B1 strain presented no differences in growth rate, chitin content or hyphal structures when compared to the WT strain. Similar to the *An*CsmB deletion strain, the MBCA∆B1 strain manifested severely reduced growth, and exhibited balloon-shaped hyphae, brown clumps and lysis at hyphal subapical regions. Again, these results show that the MMDs fulfill similar roles in both fungi, but that, interestingly, the CSDs have different, non-overlapping functions in both CS proteins. Classes V and VII of CS, significantly differ, despite similar amino acid sequences, in the function of their CSD [115].

In *A. nidulans*, deletion of the *An*CsmB MMD resulted in a strain with reduced growth and balloon-like hyphal structures [114]. The absence of the *An*CsmB MMD inhibited precipitation of *An*CsmB CS with actin filaments, suggesting that this domain is important for CS localization (as discussed below), chitin synthesis, and interaction of this CS with actin [114]. Deletion of the *An*CsmB ORF resulted in a strain with reduced growth and abnormalities in hyphal development, where balloon-like structures in the subapical regions of hyphae were observed when compared to the WT strain. Furthermore, intrahyphal hyphae, brownish clumps, a low rate of conidiation and the presence of abnormal conidiophores were also observed in the ∆*AncsmB* strain when compared to the WT strain. The addition of the osmotic stabilizer KCl reversed some of the aforementioned phenotypes, although conidia production remained low in all conditions. Simultaneous deletion of *AncsmA* and *AncsmB* (class V and class VII) was unsuccessful, suggesting that both CSs are essential for fungal growth and development. Furthermore, duplication of *AncsmA* in the *AncsmB* deletion background, resulted in an *An*CsmA CS overexpression strain. *An*CsmA overexpression was not able to restore growth phenotypes, suggesting that the *An*CsmB CS is crucial for growth and development and that both CSs have different functions. 

In *A. fumigatus*, deletion of *AfcsmB* did not impair CS activity, cell wall chitin content or the expression of other CS-encoding genes. In contrast, the ∆*AfcsmB* strain presented decreased radial growth on solid media, with 10–20% of intrahyphal growth. Furthermore, the ∆*AfcsmB* strain produced a severely reduced number of conidia, even in the presence of an osmotic stabilizer, and conidiophores presented structural abnormalities [71]. These phenotypes were also observed for the double deletion strains ∆*AfcsmA* and ∆*AfcsmB*, with the exception that this strain presented 70–80% intrahyphal growth [71]. The cell wall of ∆*AfcsmB* conidia manifested both hydrophobic and hydrophilic features as well as exposed polyssacharides when compared to the WT strain; whereas ∆*AfcsmA* and ∆*AfcsmB* conidia had heterogenous morphological features similar to conidia from the ∆*AfcsmB* strain, with rodlets being easily damaged, suggesting a fragile and disorganized conidial structure [108].

#### 3.3.2. Deletion of Other CSs from Division 2

To understand the role of CS from division 2 in *A. fumigatus* growth and development, Muszkieta and colleagues (2014), constructed single, double, triple and quadruple CS mutant strains (∆*AfcsmA* ∆*AfcsmB* ∆*AfchsF* ∆*AfchsD* (Afu1g12600)) [103]. A reduction in radial growth that was proportional to the increase in the number of deleted genes encoding division 2 CS (e.g., ∆*AfcsmA* ∆*AfcsmB,* ∆*AfcsmA* ∆*AfcsmB* ∆*AfchsF,* ∆*AfcsmA* ∆*AfcsmB* ∆*AfchsF* ∆*AfchsD*) was observed. Hyphae of the ∆*AfcsmA*, ∆*AfcsmB*, ∆*AfcsmA* ∆*AfcsmB* ∆*AfchsF* and ∆*AfcsmA* ∆*AfcsmB* ∆*AfchsF* ∆*AfchsD* strains were balloon-shaped, and chitin distribution within the mycelial cell wall was also impaired. Division 2 CS gene deletion strains, with the exception of ∆*AfchsF* and ∆*AfchsD*, had significantly reduced conidiation, with conidiation defects being proportional to the number of deleted genes. The addition of KCl rescued the conidiation defect of the ∆*AfcsmA* ∆*AfcsmB* ∆*AfchsF* ∆*AfchsD* strain. In addition, conidia of the ∆*AfcsmA,* ∆*AfcsmB,* ∆*AfchsF,* ∆*AfchsD,* ∆*AfcsmA* ∆*AfcsmB,* ∆*AfcsmA* ∆*AfcmsB* ∆*AfchsF* and ∆*AfcsmA* ∆*AfcsmB* ∆*AfchsF* ∆*AfchsD* strains presented a loose cell wall with a layer of melanin that easily detached from the inner cell wall layer, resulting in conidia with increased hydrophilicity when compared to the parental strain. Germination of the ∆*AfcsmA* ∆*AfcsmB* ∆*AfchsF* and ∆*AfcsmA* ∆*AfcsmB* ∆*AfchsF* ∆*AfchsD* strains resulted in hyphae with abnormal chitin deposits, suggesting fragile cell walls that were more susceptible to stress conditions. All *A. fumigatus* division 2 CS deletion strains, with the exception of the ∆*AfchsF* and ∆*AfchsD* strains, presented decreased CS activity, although no significant differences in cell wall chitin content were observed, with the exception of the ∆*AfchsD* strain, which contained 125% chitin when compared to the parental strain. Conidia from the ∆*AfcsmA,* ∆*AfcsmB* and ∆*AfchsF* strains presented significantly reduced content of cell wall chitin. Furthermore, short chitin microfibrils were observed for the ∆*AfcsmA*, ∆*AfcsmB* and ∆*AfcsmA* ∆*AfcsmB* ∆*AfchsF* ∆*AfchsD* strains, possibly explaining the observed morphological abnormalities during asexual development. Additional differences in cell wall polysaccharide concentrations were also observed for the division 2 CS deletion strains. Deletion of the class VI CS ∆*AfchsD* resulted in a strain with normal hyphal morphology, conidiation, germination and chitin synthase activity, but with significantly increased amounts of β-1,3-glucan and decreased concentrations of α-1,3-glucan. In contrast, deletion of class V CS ∆*AfcsmA* resulted in a strain with reduced β-1,3-glucan concentrations and increased concentrations of α-1,3-glucan [96,103].

As chitin content was significantly reduced in the deletion strain ∆*AfchsG* (division 1) and the division 2 ∆*AfcsmA,* ∆*AfcsmB* and ∆*AfchsF* single deletion strains, Muszkieta and colleagues (2014) constructed a strain deleted for the aforementioned four genes. The resulting strain presented significantly reduced growth, with hyphae that did not grow more than 1.3 cm after 3 weeks, in addition to having balloon-shaped hyphae and increased hyphal branching when compared to the parental strain. The same strain also produced conidiophores without conidia, whereas mycelial cell wall chitin and α-1,3-glucan concentrations were significantly reduced, and β-1,3-glucan concentrations were increased. These results suggest that CS from divisions 1 and 2 cooperate in the biosynthesis of the *A. fumigatus* cell wall chitin skeleton, a structure that is important for antifungal drug resistance, growth and virulence [103].

### 3.4. Localization of CSs in Aspergillus Spp.

In agreement with the changing requirements of chitin synthesis during the different fungal developmental stages [83], CS are located at different loci within the cell as well as expressed in a growth-dependent manner. *An*ChsA and *An*ChsC are present in the metulae, phialides and conidia of *A. nidulans* [105], with the simultaneous deletion of these genes resulting in severely decreased conidial production and abnormal conidiophores. Similarly, *An*chsD is expressed in asexual structures, such as vesicles, metulae, phialide and conidiophores in *A. nidulans* [116].

*AnchsB*, which encodes a CS with 4–7 transmembrane domains, is targeted to the membrane during septa formation, and is localized at hyphal tips, conidia [107] and the Spitzenkörper (SPK) [117]. The SPK is a structure present in the hyphal apices of actively growing filaments, that dictates direction and growth, and that supplies the hyphal tip with proteins required for growth and cell-wall maintenance [118]. *An*ChsB forms clusters at the hyphal apex in close proximity to the plasma membrane; and these clusters change shape from a globular to a crescent form, representing the changes in vesicle accumulation at the SPK site to fusion with the apical plasma membrane [119].

Vesicles containing biomolecules, cell wall and cell membrane components accumulate at hyphal apices, forming the SPK, which dictates fungal cell growth dynamics [120,121,122]. Through quantitative super-resolution photoactivation localization microscopy (PALM) analysis, Zhou et al., 2018, showed that vesicles accumulate at the SPK during hyphal slow growth, and then, the vesicles fuse with the plasma membrane to elicit fast growth [119]. The speed of the vesicles containing the *An*ChsB CS-protein at the anterograde movement (from the back to the tip) was either 2–4 μm s^−1^ or 7–10 μm s^−1^. The retrograde transport (from tip to back) was less common and had a reduced speed (<~7 μm s^−1^). Analyzing the transport of vesicles containing the early endosomal marker protein GFP-RabA and the secretory vesicle (SV) marker protein mEosFPthermo (monomeric variant of EosFP, a fluorescent protein whose fluorescence changes from green to red at 390 nm)-TeaR, it was shown that early endosomes (EE) move at a slow speed (2.0 ± 0.5 μm s^−1^, with identical values for anterograde and retrograde movement), and SV move at higher speeds (7.9 ± 3.6 μm s^−1^ for anterograde movements and 8.3 ± 3.8 μm s^−1^ for retrograde movements) [119]. This suggests that the *An*ChsB CS-protein can be carried either by the EE or via a SV [119]. Furthermore, MyoV (myosin-5) was also shown to be important for the transport of *An*ChsB-containing vesicles to the hyphal tip. Considering that some *A. nidulans* proteins are transported along microtubules and actin filaments to the hyphal tips [123,124], it was suggested that the CS *An*CsmA and *An*ChsB are shuttled to the hyphal tips by kinesins, small molecular motor proteins involved in intracellular transport [117,125]. Deletion of *myoV* resulted in the absence of an accumulation of vesicles at the hyphal tip, suggesting that myosin-5 carries the CS-laden vesicles to the site of exocytosis. The absence of the kinesin UncA also inhibited *An*ChsB CS-protein accumulation at the hyphal tip, resulting in random, fast movement of this CS within the hyphae and suggesting that the *An*ChsB CS-protein may also be transported through secretory vesicles [119]. Secretion dynamics of *An*ChsB were shown to occur at hyphal apical regions via indirect endocytic recycling, where the diffused protein is internalized by subapical actin patches (an endocytosis site) and reconducted from endosomes to the trans-Golgi network cisternae in a Sec7-, GARP- (Golgi-associated retrograde protein) and Rab6-dependent manner [126]. In addition, the adaptor protein (AP)-2 complex, which is involved in the endocytosis process [127], promotes *An*ChsB internalization from the subapical collars of the hyphal surface, and is also important for *An*ChsB localization at hyphal tips [128]. 

In addition to *An*ChsB, the absence of KinA, but not UncA, resulted in the subapical localization of GFP-*An*CsmA when compared to the WT and *uncA-*deleted strains. Both GFP-*An*CsmA and GFP-*An*ChsB are transported together, with their retrograde and anterograde transport—but not velocity of movement—being dependent on KinA and UncA. In addition, the transport of GFP-*An*CsmA and GFP-*An*ChsB was abolished in a mRFP-KinA^rigor^ strain, where a point mutation in the ATP-binding domain inhibited movement along microtubules, suggesting that *An*CsmA and *An*ChsB CSs are transported to hyphal tips by KinA [117]. Furthermore, the same study showed that the KinA-dependent transport of *An*CsmA is independent of the MMD [117]. Further evidence for the interaction between *An*ChsB and *An*CsmA with mRFP-KinA^rigor^ was obtained in experiments with strains expressing FLAG-*An*ChsB mRFP-KinA^rigor^ and HA-*An*CsmA mRFP-KinA^rigor^. Through performing cellular fractionation experiments, FLAG-*An*ChsB and *An*CsmA-HA were detected in fractions that contain vesicles, Golgi structures and endosomal membranes. Furthermore, it was shown that although both *An*CsmA and *An*ChsB are transported by KinA to the growing hyphal tip, they are transported in different vesicles [117].

*An*CsmA is present at forming septa and as mobile spots in the cytoplasm and along the apical membrane [107,117]. The MMD in the *An*CsmA protein is predicted to be responsible for the localization of the CS domain, and therefore, for the correct localization of chitin synthesis [94]. Indeed, *An*CsmA-HA was shown to colocalize with actin at hyphal tips and at sites where septa are formed [129], and deletion of MMD resulted in *An*CsmA-HA localizing to large organelles and tubular structures [129]. Furthermore, *An*CsmA-HA was detected every 24 h by western blot when mycelia were grown in a complete medium for 5 days continuously, suggesting a patterned expression of this enzyme [130]. Cellular fractionation experiments showed that *An*CsmA-HA was present in low-speed pellets, suggesting that the *An*CsmA CS is an integral protein with several transmembrane domains. In agreement with the *An*CsmA transcriptional patterns, *An*CsmA-HA concentrations were higher in hyphae grown under low osmotic conditions than when compared to high osmotic conditions, further supporting a role for *An*CsmA in cell wall integrity during hyphal extension [113,130].

The class VII CS *An*CsmB localizes to hyphal tips and at forming septa, and similar to *An*CsmA and the *An*CsmB MMD, interacts with actin F. Once septa are mature, *An*CsmB disappears from the sites, suggesting that this protein, together with *An*CsmA, is essential for and has a compensatory effect on fungal cell wall integrity during growth and development [95].

### 3.5. Regulatory Pathways Controlling the Expression of CS-Encoding Genes

**Figure 4:**
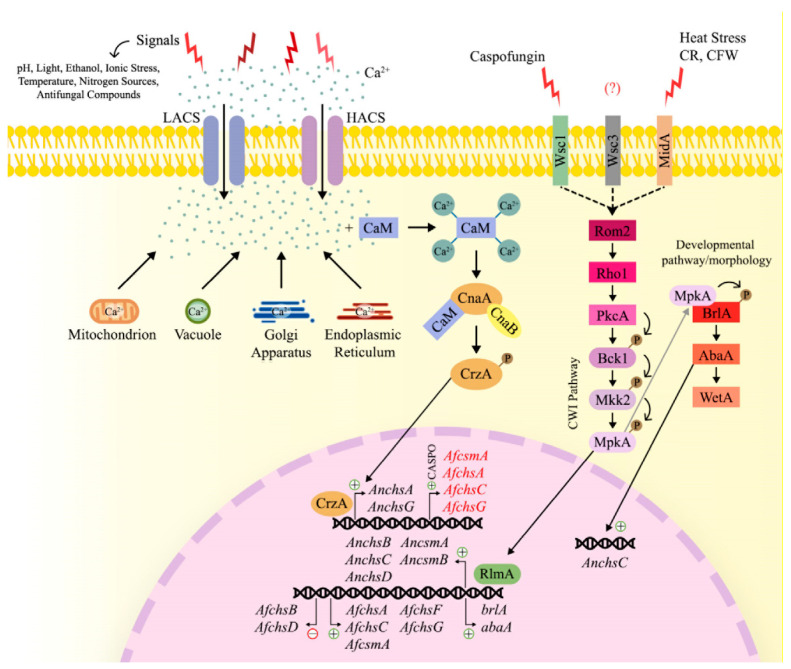
Regulation of chitin biosynthesis in response to extracellular signals and/or in the presence of stresses, including changes in pH, temperature, light, food sources and in the presence of antifungal compounds, an increase in intracellular Ca^2+^ concentrations occur. Calcium channels regulate the influx of calcium through the “High affinity Ca^2+^ influx system” (HACS), which is activated when Ca^2+^ availability is low, or the “Low affinity Ca^2+^ influx system” (LACS), when calcium availability is high. In addition, Ca^2+^ ions are also released from intracellular stores, such as mitochondria, vacuoles, the Golgi apparatus and the endoplasmic reticulum. Upon an increase in cytosolic Ca^2+^ concentrations, the protein Calmodulin (CaM) binds to 4 Ca^2+^ molecules, which triggers conformational changes in this enzyme. Subsequently, CaM is able to bind to calcineurin, a heterodimeric protein composed of catalytic (CnaA) and regulatory (CnaB) subunits, and activate it. Calcineurin, in its active form, phosphorylates the transcription factor CrzA, which subsequently translocates to the nucleus. CrzA binds to the promoter region of several *A. nidulans* chitin synthase-encoding genes, including *AnchsA* and *AnchsG*, initiating transcription of these genes. In *A. fumigatus*, the calcium-calmodulin-calcineuring signaling pathway is also activated during the caspofungin paradoxical effect (CPE), where CrzA binds to the promoter region of *AfcsmA, AfchsA, AfchsC* and *AfchsG*. In addition, the antifungal compound caspofungin, can also be sensed by the transmembrane protein Wsc1. Furthermore, MidA, an additional transmembrane sensor, is involved in the resistance against heat stress and cell wall perturbing agents, such as calcofluor white (CFW) and Congo red (CR). Wsc1 and MidA activate the small GTPase Rho1 through the interaction with Rom2 (a guanine nucleotide exchange factor). Rho1 will activate Protein Kinase C (PkcA), which in turn activates the cell wall integrity (CWI) pathway. The CWI pathway is composed of the MAPK kinase kinase Bck1, the MAPK kinase Mkk2 and the MAPK MpkA, which phosphorylate one another. As a result, MpkA translocates to the nucleus, where it interacts with the transcription factor RlmA. RlmA is involved in the expression of chitin synthase-encoding genes in *A. nidulans* and *A. fumigatus*, including *AnchsB, AnchsC, AnchsD, AncsmA, AncsmB, AfchsA, AfchsC, AfchsF, AfchsG* and *AfcsmA.* RlmA also regulates the repression of the *AfchsB* and *AfchsD* genes in these conditions. RlmA binds to the promoter regions of the genes *brlA* and *abaA*, both of which are involved in fungal developmental and conidiation processes. Furthermore, MpkA physically interacts with BrlA and AbaA regulates the expression of *AnchsC*, further supporting the notion that the regulation of chitin biosynthesis in fungal cell walls is subject to multiple pathways and the crosstalk between these pathways. Dotted lines: represent the signal transduction in response of Wsc1, Wsc3 and MidA activation.

The fungal cell wall is the first point of contact and primary interaction site for the fungus with the extracellular environment. Subsequently, external cues, such as pH variation, heat shock, osmotic stress, oxidative stress, nutrient limitation and antifungal drugs, have an effect on the cell wall and activate and/or modulate intracellular signaling pathways [131] (Figure 4). In response to these cues, the fungal cell wall is constantly being remodeled in order to withstand and counteract adverse extracellular conditions, and to ensure growth and reproduction and to avoid cell death [132]. In addition, cell wall compensatory alterations can occur in a condition-specific manner, including increased chitin and glucan biosynthesis and/or a redistribution of chitin [78].

#### 3.5.1. Intracellular Trehalose Levels

Trehalose biosynthesis is catalyzed by trehalose-6-phosphate synthase and trehalose-6-phosphate phosphatase [24]. In *S. cerevisiae,* trehalose-6-phosphate synthase (Tps1p) and trehalose-6-phosphate phosphatase (Tps2p) form a protein complex with the two regulatory subunits Tsl1p and Tps3p [133]. *A. fumigatus* has two paralogous genes for *tps1*, named *tpsA* and *tpsB* (*tpsA/B)*, one orthologous gene for *tps2*, termed *orlA*, and the two regulatory subunits TslA and TslB [133]. The trehalose regulatory subunit TslA was shown to be important for intracellular trehalose levels in mycelia and conidia, as well as for cell wall polysaccharide concentrations. The *ΔtslA* strain presented increased cell wall chitin content and CS activity, and reduced β-glucan exposure [134]. In addition, the CS *Af*CsmA physically interacts with TslA, the latter of which is important for the correct localization of this CS at hyphal tips and septa [134]. Furthermore, TslA was also shown to interact with the multifunctional RNA-binding protein SsdA. Deletion of *ssdA* resulted in a strain with significantly increased intracellular trehalose concentrations in both mycelia and conidia, whereas decreased trehalose levels were observed in a *ssdA* overexpression (OE::*ssdA*) strain [135]. Both strains had decreased growth rates. The *∆ssdA* strain had increased resistance to the cell wall perturbing agents Congo red, calcofluor white and caspofugin; whereas the OE::*ssdA* strain was highly sensitive to these agents. SsdA was shown to be important for cell wall polysaccharide concentrations, with the *∆ssdA* strain having decreased chitin levels and CS activity and the OE::*ssdA* strain having increased chitin levels and CS activity. Both the deletion and overexpression strains also presented lower levels of β-1,3-glucan in their cell wall. SsdA was also shown to be important for the correct cellular localization of CsmA, with CsmA being randomly dispersed within the hyphae in the OE::*ssdA* strain. In summary, these data suggest that intracellular levels of trehalose are important for *A. fumigatus* cell wall integrity, and that TslA functions as a chitin biosynthetic regulator in *A. fumigatus* [134,135]. This is perhaps not surprising as the type of extracellular nutrient source, in particular different carbon sources, have been shown to affect the composition and organization of the cell wall in a number of fungi.

#### 3.5.2. The Calcium-Calcineurin Signaling Pathway

In addition to the trehalose metabolic pathway, the calcineurin signaling pathway was also shown to be crucial for fungal cell wall integrity, cation homeostasis, cell cycle progression, hyphal branching, sclerotial development and pathogenesis [136] (Figure 4). Calcineurin is a Ca^2+^/calmodulin (CaM)-phosphatase-dependent heterodimeric protein that is composed of a catalytic (CnaA) and a regulatory (CnaB) subunit [137]. Extracellular signals, such as ionic stress, ethanol, light, temperature, pH, nitrogen sources and antifungal compounds, activate the plasma membrane calcium system, which can either be the high-affinity Ca^2+^ influx system (HACS) when the availability of calcium is low, or the low-affinity Ca^2+^ influx system (LACS) when Ca^2+^ availability is high [138]. The release of calcium from intracellular storages, such as vacuoles, the endoplasmic reticulum, mitochondrion and Golgi apparatus, results in an increase in intracellular Ca^2+^ concentrations [139]. The increased intracellular calcium concentration is sensed by calmodulin, which binds 4 Ca^2+^ ions [136]. As a consequence, calmodulin undergoes a conformational change and binds to calcineurin. Calcineurin is now in its active form and dephosphorylates the transcription factor CrzA, which will translocate to the nucleus and bind to the target promoters of approximately 102 genes. These genes encode enzymes involved in transport, regulation of biological processes, response to stress, chemicals and developmental processes, and the transcriptional response modulates the cellular response to the extracellular cue [140,141]. The predicted CrzA DNA bindings consensus sequence— (G(T/G)GGC(T/A)G(T/G)G)—is present in the promoter regions of the CS genes *AnchsC* and *AnchsG* (AN1046), suggesting a possible transcriptional regulations of these two CS by CrzA in *A. nidulans* [142].

Further evidence for the involvement of calcium signaling in cell wall integrity was obtained from studies in *A. fumigatus*. Deletion of *cnaA* in this fungus, resulted in a strain with severe defects in growth, filamentation, conidia production and morphology [143]. Furthermore, the *A. fumigatus* Δ*cnaA* mutant strain presented a reduced β-glucan hyphal content, when compared to the WT strain, and this decrease is even greater when the Δ*cnaA* strain is treated with caspofungin, a β-1,3-glucan synthase inhibitor. In contrast, the addition of the CS inhibitor Nikkomycin Z leads to a compensatory increase in β-glucan levels in the Δ*cnaA* strain. Indeed, transcriptional expression of the CS-encoding genes *AfchsA*, *AfchsC*, *AfcsmA*, *AfchsF* and *AfchsG* is significantly reduced in the Δ*cnaA* strain [144]. Similar defects in growth, cell wall architecture and septa formation were also observed for the Δ*cnaB* and Δ*cnaA* Δ*cnaB* strains. In addition, a ~ 40% reduction in β-glucan content was observed in the Δ*cnaA*, Δ*cnaB* and Δ*cnaA* Δ*cnaB* strains when compared to WT strain. Furthermore, chitin content was increased by ~40% in the Δ*cnaA* Δ*cnaB* strain and by ~20% in the single deletion strains when compared to the WT strain, showing a compensatory effect. Despite the observed increase in chitin content in these strains, the expression of *AfchsA*, *AfchsB*, *AfchsC*, *AfchsD*, *AfcsmA*, *AfchsF*, *AfchsG* and *AfcsmB* was downregulated in the Δ*cnaB* and Δ*cnaA* Δ*cnaB* strains. These observations suggest that possibly an abnormal assembly of cell wall components in the calcineurin deficient strains is occurring, with a likely deficient incorporation of chitin into the cell wall, due to a decreased β-glucan content [145].

In agreement with the observed reduced levels of β-glucan in the Δ*cnaA* strain, Cramer and colleagues (2008) also showed a decreased expression of *fksA* (-2.1-fold expression change) in this strain [144]. The gene *fksA* codifies for the catalytic subunit of the β(1,3)-glucan synthase complex, that together with the regulatory subunit Rho1 are responsible for biosynthesising β-glucan. FksA is localized to the hyphal apex in proximity to Rho1, a GTP-ase that is highly expressed in the Δ*cnaA* (10,9-fold expression change) strain when compared to the WT strain [144,146]. Interestingly, Rho1 can elicit cell wall composition via activation of FksA, and also, this GTP-ase is a component of the cell wall integrity (CWI) pathway [147,148,149].

#### 3.5.3. The Cell Wall Integrity (CWI) Pathway

The CWI pathway is an intracellular signaling cascade, which responds to various environmental stimuli that perturb the cell wall and membrane homeostasis; this signaling pathway modulates gene expression to keep the integrity of the fungal cell wall intact [131,150]. In *A. fumigatus*, cell wall abnormalities are detected by sensor transmembrane proteins, namely Wsc1, Wsc3 and MidA (Figure 4). Wsc1 is required for resistance against the antifungal agent caspofungin, whereas MidA is involved in resistance to heat stress and chitin binding agents, such as Congo red and CFW. All three proteins have overlapping functions, as abnormalities in these sensor proteins result in defects in growth and conidiation [148]. Once cell wall stress activates a given transmembrane sensor, the small GTPase Rho1 is activated through interaction with the guanine nucleotide exchange factor (GEF) Rom2, which is localized between the sensor and Rho1 [149]. Subsequently, Rho1 activates the protein kinase C PkcA. When the *A. fumigatus* CEA17 WT strain was treated with the β-glucan-intercalating agent Congo red, the expression of genes *pkcA, mpkA, rlmA, fksA* was highly induced. Similarly, the CS-encoding genes *AfchsA*, *AfchsC*, *AfcsmA*, *AfchsF*, *AfchsG* had increased expression in response to Congo red. PkcA has been shown to be important for cell wall maintenance in the presence of Congo Red, as it regulates CS-encoding genes [151]. Using a strain that harbors a Gly579Arg substitution in the PkcA C1B domain, Rocha and colleagues, 2015, demonstrated a higher sensitivity of this mutant strain to cell wall perturbing agents (Congo red, CFW, anidulafungin, SDS and caffeine), nikkomycin Z and fluconazole [151]. The most prominent growth effects observed for the *pkcA^G579R^* mutant strain, were for Congo red and CFW. In the WT strain, a time-dependent increase in MpkA phosphorylation was observed in the presence of Congo red; this phenomenon was not observed for the *pkcA^G579R^* strain, where the presence of Congo red, resulted in reduced *mpkA* expression and MpkA phosphorylation. The expression of *rlmA,* coding for a transcription factor, was also decreased in the mutant strain in the presence of Congo red, suggesting the involvement of PkcA-MpkA in RlmA activation in the presence of Congo red. The authors also showed that Congo red increases the expression of CS genes (*AfchsA, AfchsC, AfchsF, AfchsG and AfcsmA*) in the WT strain, which was not seen in the *pkcA^G579R^* strain. After Congo red exposure, the expression of *AfchsA, AfchsC, AfchsF, AfchsG and AfcsmA* were reduced in the mutant strain. In contrast, this was not observed for the expression of *AfchsB, AfchsD* and *AfcsmB*. In the WT strain, the presence of Congo red resulted in the repression of *AfchsB* and *AfchsD*. In the *pkcA^G579R^* strain, before the addition of Congo red, the expression of *AfchsB* and *AfchsD* was higher than when compared to the WT; after incubation in the presence of Congo red, the expression levels of these CS were decreased, suggesting a possibly compensation mechanism for the absence of PkcA. Furthermore, the expression of *AfcsmB*, was also not modulated in the presence of Congo red in the WT and mutant strains [151]. Altogether, these data suggest the importance of PkcA for CS gene expression.

The deletion of MpkA and RlmA, in the presence of Congo red resulted in the transcriptional regulation of the genes *fksA, mpkA, rlmA, AfchsA*, *AfchsC*, *AfcsmA*, *AfchsF* and *AfchsG* by RlmA and MpkA. Furthermore, the same study showed that *pkcA* was negatively regulated by RlmA and MpkA, and *AfcsmB* was positively regulated by RlmA and negatively by MpkA. The CS-encoding genes *AfchsB* and *AfchsD* were both negatively regulated by RlmA, and *AfchsD* was positively regulated by MpkA in the presence of Congo red [152]. These studies suggest a complex interplay between PkcA, RlmA and MpkA in regulating cell wall maintenance in *A. fumigatus*. 

Similar to *A. fumigatus*, PkcA was also involved in the regulation of CS-encoding genes in *A. nidulans*. Katayama et al., 2014, constructed a strain where PkcA was ectopically activated through the substitution of PkcA Arg429 to alanine and placed under the control of the *alcA* promoter (R429A-1 strain). When R429A-1 was transferred from repressing to inducing conditions, a significant increase in the mRNA levels of the CS-encoding genes *AnchsB*, *AnchsC*, *AnchsD*, *AncsmA* and *AncsmB* but not *AnchsG* was observed, suggesting that PkcA is involved in the regulation of these genes [142]. 

Protein kinase C activates the cell wall integrity (CWI) pathway, which consists of a mitogen-activated protein kinase (MAPKs) cascade. In the CWI pathway, this cascade is composed of the MAPK kinase kinase Bck1, the MAPK kinase Mkk2 and the MAPK MpkA, which phosphorylate one another [153]. Upon phosphorylation, MpkA translocates to the nucleus, where it activates the transcription factor RlmA, which subsequently transcriptionally upregulates cell wall biosynthesis genes and tolerance to oxidative stress [152]. To determine whether the expression of CS-encoding genes is directly regulated by PkcA or by downstream components, such as RlmA; Katayama et al., 2015, constructed the Δ*rlmA-1* (*rlmA* deleted) and R429AΔ*rlmA-1* [*pkcA*(R429A) under the control of the *alcA* promoter in addition to a wild type *pkcA* copy, and also with a deleted *rlmA* gene strains. The expression of *AnchsB, AnchsC, AnchsD, AncsmA* and *AncsmB* is mainly regulated by RlmA, as the mRNA levels of these genes are similar between the Δ*rlmA-1* and R429AΔ*rlmA-1* strains when shifting from repressing to inducing conditions. The authors suggested that constitutively activated PkcA leads to the higher expression of some CS-encoding genes through the activation of the CWI pathway and the TF RlmA [142]. In contrast, the *AnchsG* mRNA levels were lower in the R429AΔ*rlmA-1* strain when compared to Δ*rlmA-1* strain in the same conditions, suggesting that the expression of *AnchsG* is independent of RlmA [142]. Similarly, the expression of *AnchsC* also occurs in a RlmA-independent manner, as *AnchsC* mRNA levels were higher in the R429AΔ*rlmA-1* strain, when compared to the Δ*rlmA-1* strain [142].

The *A. fumigatus* Δ*rlmA* strain has a decrease in conidia production, and exhibits undifferentiated aerial hyphae formation, suggesting that RlmA is important for conidiation [152]. A delay in conidial formation was also observed for the Δ*rlmA* strain, as well as for the Δ*pkcA^G579R^* and Δ*mpkA* strains, which was accompanied by reduced conidial hydrophobicity. When analyzing chitin content in these mutants, it was observed that the *ΔpkcA^G579R^* strain had similar chitin levels as the WT strain, whereas the Δ*mpkA* and Δ*rlmA* strains had lower and higher chitin levels, respectively, when compared to the parental strains. All these data highlight the relevance of the CWI pathway for the initial steps of conidiation [154].

#### 3.5.4. The Developmental Signaling Pathway

In *Aspergillus* species, conidiation is regulated by a developmental signaling pathway. The central regulators of conidiation constitute of BrlA, AbaA and WetA [155] (Figure 4). BrlA is involved in the initiation of conidiation, as it is expressed at the early stages of conidia production. Additionally, BrlA is important in the initial steps of conidiophore formation, and this transcriptional regulator is necessary for the expression of other conidiation-related genes including *abaA, wetA, vosA and rodA* [156,157]. BrlA also transcriptionally induces *abaA*, whose function is related to the differentiation and functionality of phialide development during conidiophore maturation [156]. The *abaA* gene codifies for a protein with a DNA-binding motif, ATTS sequence/TEA and a leucine zipper [158]. In addition, AbaA binds to the consensus sequence 5′-CATTCY-3′, termed the “AbaA response element” (ARE), that is present in the promoter region of several genes, including *brlA, wetA, rodA* and *abaA* [155,159]. The gene *wetA* is activated by AbaA, and is responsible for the final steps of conidiation, including the synthesis of the C4 inner layer of the conidia cell wall, which is important for the maturation and impermeability of conidia [156,157]. In addition, WetA is important for conidia trehalose production, conidia germination and early steps of fungal growth [156,157].

An interesting crosstalk between the CWI pathway and the central regulators of asexual development in *A. fumigatus* were described by Rocha and colleagues, 2020 [154]. The expression of genes coding for central regulators of asexual development (*brlA, abaA* and *wetA*) was also increased in the WT strain during conidiation, whereas this expression was absent in the CWI pathway mutants (*pkcA^G579R^,* Δ*mpkA*, Δ*rlmA*), corroborating the conidiation abnormalities observed in these mutant strains. Furthermore, RlmA was shown to bind in vivo to the promoter regions of *brlA* and *abaA*, and the authors also found that MpkA physically interacts with BrlA, suggesting an important role of the CWI pathway during conidiation in *A. fumigatus* [154].

In *A. nidulans*, Park and colleagues, 2003, demonstrated that AbaA was able to bind to, at least in vitro, to three putative AREs in the promoter region of the CS-encoding gene *AnchsC*, and in vivo assays demonstrated that AbaA directly activates transcription of *AnchsC* [160]. In agreement, expression of *AnchsC* is reduced in the *brlA* and *abaA* deletion strains, reinforcing the participation of AbaA in the regulation of chitin synthesis during conidiation [106,160]. Furthermore, expression of *brlA* and *abaA* was observed in the WT, ∆*AnchsA* and ∆*AnchsC* strains during conidiophore development, whereas this transcriptional upregulation is lost in the ∆*AnchsA* ∆*AnchsC* double mutant strain [106], suggesting that *brlA* and *abaA* are important for *A. nidulans* conidiophore development.

In *A. nidulans*, extracellular cues, such as heat and osmotic stress, activate the aforementioned signaling and regulatory pathways, resulting in the transcriptional induction of CS-encoding genes in a cue-dependent manner. This further highlights the importance of cell wall dynamics and integrity for fungal survival and withstanding different stresses. The expression of *AnchsA* was increased during conidiophore development and the GFP-tagged *AnchsA* protein was observed in asexual structures. Osmotic stress and sodium acetate also increased the expression of this CS-encoding gene, whereas heat stress and different carbon sources did not influence the transcriptional expression of *AnchsA*. Expression of *AnchsB* was shown to be ubiquitous in the fungus and less dependent on the fungal developmental stage. Similar to *AnchsA, AnchsB* is transcriptionally induced in the presence of higher concentrations of sodium acetate, but not in the presence of osmotic or heat stress or in the presence of simple sugars. The expression of *AnchsC* was highly dependent on the hyphal developmental stage and transcriptional expression was observed in vegetative mycelia and during sexual differentiation. Furthermore, *AnchsC* was transcriptionally induced in the presence of osmotic stress and sodium acetate but not when the fungus was exposed to heat stress and simple sugars. Taken together, these data suggest that the expression of CS-encoding genes is dependent on the fungal developmental stage and also responds to different stresses as well as the presence of growth-stimulating carbon sources in a signal-specific manner [161]. To further investigate the effect of heat stress on CS gene expression in *A. nidulans*, Katayama and colleagues, 2014, constructed a temperature-sensitive *pkcA* mutant strain (*pkcA-*ts), where PkcA is inactivated at restrictive temperatures (42 °C). When strains were grown at the permissive temperature of 20 °C and then shifted to the restrictive temperature, the mRNA levels of *AnchsA*, *AnchsF*, *AncsmA* and *AncsmB* significantly increased in the WT strain, whereas expression of *AncsmA* and *AncsmB* did not occur in the *pkcA*-ts strain. In summary, these data suggest that, in the presence of heat stress, PkcA is involved in the regulation of several CS-encoding genes [142]. 

Together, the aforementioned studies highlight the complex regulatory network in which CS-encoding genes are embedded, and how the expression of these genes is highly variable and dependent on defined conditions, such as fungal developmental stage, temperature shifts and the presence of extracellular cell walls and cell membrane-damaging agents [79].

## 4. Chitin Biosynthesis as a Target for Antifungal Drugs

Opportunistic fungal infections, including those caused by *Aspergillus* spp. have become a major global health problem for both immunocompetent and immunocompromised patients. It is estimated that invasive fungal infections result in over 1.5 million deaths per year, which is higher than patients dying globally from breast cancer or malaria [162]. The fungal cell wall, including chitin synthesis, presents an interesting target for antifungal drug development, simply because humans do not have a cell wall [1]. Indeed, inhibition of chitin synthesis by these antifungal agents, negatively affects cell wall architecture and fungal morphology, subsequently resulting in reduced fungal viability and survival [2,162].

Nikkomycins and polyoxins are the most studied inhibitors of chitin synthesis. They are pyrimidine nucleosides linked to a di- or tri-peptide moiety, with a similar structure to the CS substrate UDP-GlcNAc. Thus, they function as competitive inhibitors, binding to the catalytic site of CS with more affinity than UDP-GlcNAc [163,164]. 

There are several types of nikkomycins: nikkomycin D, I, J, X and Z, that differ from each other mainly by the nucleoside moiety [164,165]. In vitro, nikkomycin X and Z were effective against the dimorphic fungi *Coccidioides immitis, Blastomyces dermatidis*, but were less effective against *C. albicans, C. tropicalis, Fusarium oxysporum* and ineffective against *A. fumigatus, A. flavus, A. niger, A. versicolor* and *A. nidulans* [166,167,168]. In in vivo murine models, nikkomycin Z was more effective than nikkomycin X, and was able to almost eradicate coccidioidomycosis infection, leading to increased mouse survival during blastomycosis and histoplasmosis infection [166,169]. Nikkomycin Z went to phase II of clinical trials to be used as a commercial antifungal drug against coccidiomycosis, but no further results were published [170].

The synergism between nikkomycin Z with other antifungals agents in so-called combination therapies has been investigated intensely. Li and Rinaldi, 1999, analyzed the in vitro synergistic effect of nikkomycin Z and the ergosterol biosynthesis inhibitors itraconazole or fluconazole for 110 fungal species, and found increased antifungal efficacy against *C. albicans, C. parapsilosis, C. neoformans and C. immitis* when these antifungal compounds were used in combination than when compared to single drug use only. They also observed a synergism for the combination of nikkomycin Z and itraconazole against *A. fumigatus* and *A. flavus* [168]. In vitro synergism was also observed for nikkomycin Z in combination with the echinocandins micafungin and caspofungin against *A. fumigatus*. Echinocandins are antifungal agents that inhibit β-1,3-glucan synthase, which synthesizes cell wall β-glucan. Indeed, disruption of one cell wall polysaccharide can have profound effects on the structure and arrangement of other cell wall polysaccharides. Verwer and colleagues, 2012, observed that *A. fumigatus* treated with >0.5 µg/mL of nikkomycin Z presented a high β-glucan content and a decreased chitin content at nikkomycin concentrations of 16 µg/mL. Caspofungin at 4 µg/mL significantly reduced β-glucan levels, whereas higher concentrations of 32 µg/mL resulted in increased cell wall chitin content. *A. fumigatus* strains treated with the combination of two drugs (0.125 µg/mL of caspofungin + 2 µg/mL of nikkomycin Z) presented a significant decrease in β-glucan content, and increased chitin levels [171], suggesting that a compensatory effect occurs when the synthesis of chitin or β-glucan are blocked. Antifungal drug synergism against *A. fumigatus* was not observed for polyoxin D in combination with echinocandins or the cell membrane ergosterol biosynthesis targeting drugs polyenes and triazoles [171,172,173].

Plagiochin E (PLE), a macrocyclic *bis* (bibenzyls) compound, is isolated from the liverwort plant *Marchantia polymorpha* L., and presents antifungal properties. In *Candida albicans*, PLE causes alteration in the FCW, with decreased activity of CS in vitro, inhibition of chitin synthesis in situ, decreased expression of *chs1*, and increased expression of *chs2* and *chs3* [174]. In addition, PLE causes reactive oxygen species (ROS) accumulation in *C. albicans.* ROS function as mediators of apoptosis, as *C. albicans* treated with PLE undergoes apoptosis through the activation of metacaspases [175,176]. PLE shows synergism with fluconazole against a fluconazole-resistant *C. albicans* strain, and the mechanism of this synergism is through the inhibition of efflux pumps by PLE, resulting in increased intracellular concentrations of fluconazole in *C. albicans* [177]. Despite these promising properties, to date, there are no studies testing PLE on *Aspergillus* species. Fortunately, several antifungal compounds, including some with novel antifungal mechanisms, are currently in phases II and III of clinical trials with promise to be commercially available soon [178]. These compounds do not target chitin biosynthesis and will therefore not be reviewed here.

Furthermore, CS confer resistance to antifungal agents and cell wall targeting compounds. Mellado et al., 2003, showed that the *A. fumigatus* strain simultaneously deleted for *AfcsmA* and *AfchsG*, and the single *AfchsG* deletion strain, are more susceptible to the echinocandin LY303,336. These strains exhibited significantly reduced chitin synthase activity and chitin content when compared to the parental strain [109]. In contrast, deletion of *AfchsG*, and a quadruple deletion of genes encoding CS from division 1 (*AfchsA AfchsC AfchsB AfchsG*) resulted in strains that were resistant to Congo red, Calcofluor white and nikkomycin Z, with these strains containing less β-1,3-glucan and high α-glucan concentrations in their cell wall. A compensatory effect in cell wall glucan biosynthesis therefore occurred. Deletion of the division 2 CS *AfchsD* resulted in increased β-1,3-glucan levels and decreased concentrations of α-1,3-glucan. With the exception of the *ΔAfchsF* and *ΔAfchsD* strains, all division 2 CS deleted strains were resistant to itraconazole, voriconazole and nikkomycin Z, but sensitive to Congo red and Calcofluor white when compared to the WT strain, suggesting an impairment in cell wall permeability [103].

### 4.1. The Caspofungin Paradoxical Effect (CPE)

The CPE is probably the most studied cell wall polysaccharide compensatory effect. The CPE is an event observed in some strains of *Aspergillus* and *Candida* species, whereby increased concentrations of caspofungin, in contrast to lower concentrations, are not fungicidal/fungistatic, but rather result in improved fungal growth [179,180,181]. The occurrence of the paradoxical effect (PE) is a strain- and echinocandin-dependent trait, as five out of seven *A. fumigatus* clinical isolates presented paradoxical growth in the presence of caspofungin only, but not in the presence of micafungin and anidulafungin [182]. Similarly, the routinely used *A. fumigatus* wild-type strain Af293, presents a compensatory growth effect in the presence of caspofugin, but not when grown in the presence of the echinocandins micafungin and anidulafungin. In addition to *A. fumigatus*, the PE was also observed in *A. flavus* and *A. niger* in the presence of caspofungin and in *A. terreus* in the presence of caspofungin and micafungin [183,184,185]. One mechanism through which the CPE is predicted to occur, is through an increase in cell wall chitin content when β-1,3-glucan content is decreased by the echinocandin [171,186]. Indeed, in the presence of caspofungin (0.2 or 2 µg/mL), several genes involved in cell wall biosynthesis are induced, such as CS-encoding genes (*AfchsA* and *AfchsG*), α-glucan synthase-encoding genes (*ags1* and *ags3*), as well as genes coding for β-glucan synthase (*fks1*) and other enzymes in the *A. fumigatus* CEA17 and AF293 strains [187]. The ∆*AfchsA,* ∆*AfchsC* and ∆*AfchsA* ∆*AfchsC* strains showed increased cell wall chitin content similar to the WT strain in the presence of caspofungin, and compensatory increased transcriptional expression of other CS-encoding genes, such as *AfchsA, AfchsB, AfchsD, AfcsmA, AfchsF* and *AfchsG* was not observed. These results suggest that the increase in cell wall chitin concentrations is likely due to additional post-transcriptional downstream events [188] or that other mechanisms not related to the increased cell wall chitin exist. Furthermore, the class III CS AfChsG plays a critical role in the response of *A. fumigatus* to caspofungin. In contrast to strains deleted for CS from classes II and IV, the ∆*AfchsG* strain did not show compensatory chitin content production in response to the presence of caspofugin. In addition, the ∆*AfchsC* ∆*AfchsG* strain was highly susceptible to caspofungin, even in the presence of CaCl_2_ and CFW, suggesting that AfChsG is the main CS that responds to the presence of caspofungin [189].

#### 4.1.1. Signaling Pathways Involved in the CPE

Several signaling pathways, and the crosstalk between them, have been shown to be involved in establishing the CPE. The calcium-calcineurin signaling pathways is involved in the CPE, with the ∆*cnaA* strain not showing a compensatory increase in cell wall chitin upon the addition of high concentrations of caspofungin [190]. Indeed, increased expression of the CS-encoding genes *AfchsA* (CS of class I) and *AfchsC* (CS of class III) in the presence of caspofungin was not observed for the ∆*cnaA* strain in comparison to the WT strain [182]. The expression of other CS-encoding genes, including *AfchsD, AfcsmA, AfchsF* and *AfchsG* was also increased in the WT strain when compared to the ∆*cnaA* strain, although this increase was not statistically significant, suggesting that two CS-genes are under the regulatory control of the calcium-calcineurin signaling pathway [182]. 

In the presence of the CPE (4 µg/mL of caspofungin), an increase in cytoplasmic Ca^2+^ concentrations occurs in the *A. fumigatus* CEA10 strain, together with an increased expression of calmodulin (*cmdA*) and calcineurin A (*cnaA*) genes, suggesting an activation of the calcium-calmodulin pathway [191], resulting in the activation of the transcription factor CrzA. Ries et al., 2017 showed that CrzA binds to the promoter regions of *AfchsA, AfchsC, AfchsG* and *AfcsmA* in the presence of the CPE [192]. CrzA binds to the CG [GCC(A/T)C] motif in the promoter regions of approximately 600 genes, including *AfchsA* and the β-1,3-glucan synthase-encoding gene *fks1*, in the presence of 2 µg/mL caspofungin [187]. These results provide further evidence for the involvement of the calcineurin signaling pathway in the CPE. Indeed, deletion of *crzA* resulted in a strain with a significantly thicker cell wall and increased concentrations of cell wall GlcNAc, suggesting that CrzA does not only regulate the expression of CS-encoding genes during the CPE, but is also required for cell wall organization in the absence of caspofungin. In addition, the transcription factor ZipD, which is upregulated in the presence of calcium and negatively regulated by calcineurin, was also shown to be important for the CPE. Deletion of *zipD* resulted in a strain with increased sensitivity to caspofungin, a decrease in the CPE and in the expression of CS-encoding genes. ZipD translocated to the nucleus in the presence of calcium or caspofungin in a calcineurin-dependent manner, suggesting that ZipD is involved in regulating the CPE via the calcium-calcineurin signaling pathway, although the mechanism underlying this regulation remains subject to future investigations [192]. Not surprisingly, ZipD was also associated with correct cell wall organization and composition in *A. fumigatus* [193]. The ∆*zipD* strain was sensitive to cell wall-perturbing compounds, such as Congo red and calcofluor white, had lower levels of cell wall β-1,3-glucan, higher chitin levels and a thicker cell wall when compared to the WT strain [193].

In addition to the calcineurin pathway, the CWI pathway was also shown to be important for the *A. fumigatus* CPE, with both pathways being subject to crosstalk [192]. Colabardini and colleagues, 2021, showed that the deletion of *crzA* in *A. fumigatus* (CEA17 [Δ*crzA*^CEA17^] and Af293 [Δ*crzA*^Af293^] strains) resulted in decreased MpkA phosphorylation in both *crzA* deletion background strains in the presence of most tested caspofungin concentrations. This study suggests that CrzA is important for MpkA activation, but not essential [187]. In the CEA17 WT strain, the presence of fungistatic concentrations of caspofungin resulted in increased phosphorylation of MpkA and, thus, the activation of the CWI pathway and establishment of the CPE. Increasing concentrations of caspofungin also caused transcriptional upregulation of the CS genes *AfchsA, AfchsC, AfchsG* and *AfcsmB*. Deletion of *mpkA* and *rlmA* resulted in the loss of the CPE and a differential expression pattern of CS-encoding genes. In the ∆*mpkA* strain, *AfchsA, AfchsB, AfchsC, AfchsD, AfcsmA, AfchsG* and *AfcsmB* expression was significantly increased in the presence of caspofungin, when compared to the WT strain. In the *∆rlmA* strain, expression of *AfcsmA* and *AfchsG* was increased in response to caspofungin, whereas expression of *AfchsC* was decreased during the CPE [192].

#### 4.1.2. Additional Factors Involved in the CPE

In addition to ZipD, Valero et al., 2020, showed that eleven additional transcription factors (TFs) are involved in the CPE. Strains deleted for the TFs *cbfA, nctA, nctB, nctC, fhdA, znfA, atfA, rlmA, zfpA,* AFUB_054000 and *zipD* were sensitive to caspofungin concentrations at which the CPE is usually observed in the WT strain. All these strains were also sensitive to lower concentrations (0.5 µg/mL) of caspofungin, with the exception of *ΔnctA* and *ΔrlmA*, suggesting that a correlation between reduced CPE and caspofungin resistance exists [194]. The NctA and NctB (negative factors two A and B, respectively) are transcription factors that are members of the C-repeat binding factor (CBF)/nuclear factor Y (NF-Y) family of regulators. They are part of the same transcription regulatory complex (NCT), that act as negative regulators of ergosterol biosynthesis, and are involved in the transcription of the ABC transporter CDR1B-encoding gene, which was shown to play a role in azole resistance. Deletion of *A. fumigatus nctA* and *nctB* resulted in a strain with increased resistance to various azoles (itraconazole, voriconazole, posaconazole), amphotericin B and terbinafine. In contrast, the same strain was highly sensitive to echinocandins (caspofungin and micafungin), CFW and Congo red [195]. These results highlight the importance of these TFs for mediating the *A. fumigatus* response to antifungal agents. NctC and CbfA are also members of the CBF/NF-Y TF family, proteins that are important for the mitochondrial electron transport chain [196]. NctC and CbfA were also important for fungal growth, secondary metabolite production, with the deletion of *nctC* resulting in an avirulent *A. fumigatus* strain, in a neutropenic mice model [194]. ZnfA is a TF with a zinc-finger associated domain and AtfA is a leucine zipper TF. Deletion of *atfA* in *A. fumigatus* resulted in a strain with conidia that were highly sensitive to heat and oxidative stress, contained less intracellular trehalose levels and were delayed in germination [197]. Furthermore, AtfA was shown to positively regulate genes important for oxidative stress resistance (e.g., catalases), and to negatively regulate genes encoding enzymes involved in germination (e.g., *calA* and *calB*), suggesting a role for AtfA in mediating conidial dormancy [198]. The expression of the TF ZfpA is regulated by CrzA and induced in the presence of voriconazole and calcium [140,199]. The role of the TF AFUB_05400 has not been described and does not have an identity with any described TF [194].

The *fdhA* gene encodes a TF containing a fork-head domain, which was shown to be important for cell wall stress tolerance, as the corresponding deletion strain was sensitive to Congo red, calcofluor white and high temperatures. Through performing transcriptional analyses of the Δ*fhdA* and WT strains in the presence of caspofungin, Valero et al., 2020, observed an induction of genes related to iron metabolism. The growth of the *ΔfhdA* strain in iron-limiting conditions resulted in increased sensitivity to caspofungin and a reduced CPE. This is in contrast to growth in the presence of iron-rich medium, where the CPE took place, suggesting that the homeostasis of iron is important for the resistance of *A. fumigatus* to caspofungin [194]. Indeed, Colabardini and colleagues, 2021, demonstrated that iron depletion is essential for the CPE in the *A. fumigatus* Af239 strain, as growth of Af239 in minimal media without iron (MM-Fe) and supplemented with 8 µg/mL of caspofungin was significantly reduced when compared to iron rich media and caspofungin. Furthermore, the presence of 2 µg/mL caspofungin induced the expression of the biosynthetic gene cluster (BGC) 9 in Af239. BGC9 contains genes that code for enzymes involved in the synthesis of the iron chelator hexadehydroastechrome (HAS). Overexpression of *hasA* completely abolished the CPE, suggesting that the iron homeostasis is important during the CPE in *A. fumigatus* [187].

Caspofungin inhibits β-1,3-glucan synthesis through noncompetitive binding to Fks1, a subunit of β-1,3-glucan synthase [200]. It was shown that the expression of *fks1* is also important for the establishment of the CPE [201]. To further investigate this, a conditional mutant *fks1_tetOn_* was constructed, where the *fks1* promoter region was replaced by the doxycycline-inducible Tet-On promoter. During repressing conditions (absence of doxycycline), the CPE in the presence of 8 μg/mL of caspofungin was not observed in this conditional mutant. In addition, prolonged exposure to caspofungin also did not induce paradoxical growth of this strain in the presence of caspofungin, suggesting the requirement of Fks1 for the establishment of the CPE. Furthermore, the expression of *fks1* was shown to correlate with fungal sensitivity to caspofugin, where low *fks1* expression led to higher resistance against caspofungin, and high *fks1* expression resulted in increased sensitivity to caspofungin [201].

## 5. Chitin and Host Immune System Interaction

The fungal cell wall is the interface and first point of contact between the fungal cell and the host immune cells. The fungal cell wall contains a myriad of pathogen associated molecular patterns (PAMPs), molecules containing conserved motifs that are associated with pathogen infection and that serve as ligands for immune cell receptors. Thus, PAMPs stimulate a host-specific immune response directed at eliminating the pathogen from the host. Chitin is a major fungal PAMP and the ability of chitin to induce an immune response relies on several factors, such as its source, size, and concentration [202,203,204]. Chitin can interact with pattern recognition receptors (PRRs) present on immune cells and is recognized by TLR2, Dectin-1 and mannose receptors (MR). This interaction can lead to fungal phagocytosis and cytokine secretion [12,203,204]. 

**Figure 5:**
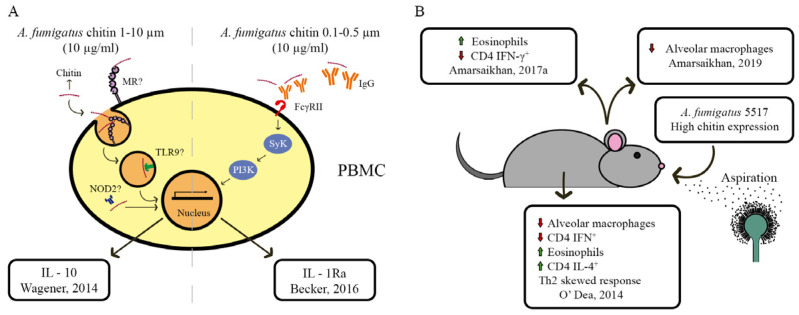
Chitin is important for the recognition of fungal pathogens by the mammalian immune system. (**A**) *A. fumigatus* purified chitin and its interaction with human PBMCs. Human PBMCs showed an increase in IL-10 secretion upon stimulation with *A. fumigatus* chitin, although this was not statistically significant. Furthermore, the receptor that mediates the increase in IL-10 upon stimulation with purified chitin, remains to be determined. Instead, when *A. fumigatus* chitin was opsonized with IgG, production of the anti-inflammatory cytokine IL-1Ra was observed. Subsequently, the *A. fumigatus* chitin-IgG complex activates the PBMC Fc-γ receptors, resulting in the activation of the Syk and phosphatidylinositol 3-kinase (PI3K) kinases and cytokine secretion. PBMC: peripheral blood mononuclear cells; IL-: Interleukin; MR: mannose receptor; TLR: toll-like receptor 9; NOD2: nucleotide-binding oligomerization domain containing 2. (**B**) Mice that aspired conidia of an *A. fumigatus* high chitin-containing strain (Af5517) displayed an increased Th2-mediated response in BALF. Indeed, inhaled Af5517 conidia led to increased eosinophils and CD4 IL-4+ T cells in BALF, while alveolar macrophages and CD4 IFN+ showed reduced numbers. BALF: bronchoalveolar lavage fluid [205,206,210,211,212].

Studies using purified *A. fumigatus* chitin have shown that this type of chitin elicits an anti-inflammatory response in vitro (Figure 5A). Incubation of *A. fumigatus* chitin (strain 237) with human peripheral blood mononuclear cells (PBMCs) stimulated the secretion of IL-10, but not TNF- α, although no statistical difference was observed when compared to unstimulated cells [205]. In another study, purified *A. fumigatus* chitin (strain CEA17_Δ*akuB*^KU80^) did not stimulate human PBMCs to produce proinflammatory cytokines (IL-1β, TNF-α or IL-6) or IL-10 [206]. In contrast, *A. fumigatus* chitin stimulated the secretion of IL-1 receptor antagonist (IL-1Ra), an anti-inflammatory cytokine. The production of IL-1Ra by human PBMCs was dependent on the opsonization of chitin by serum IgG, which in turn interacted with membrane-bound Fc-γ receptors resulting in Syk kinase and phosphatidylinositol 3-kinase (PI3K) activation [206]. Interestingly, the observed effect was Dectin-1, TLR2, TLR4, MR and NOD2-independent.

Additional studies, which were carried out in a more realistic setting and included the presence of other cell walls associated with PAMPs that showed an increased in proinflammatory response. Chitin synergizes with muramyl dipeptide (MDP), Pam3ys and LPS to induce the production and secretion of proinflammatory mediators, such as IL-1β and TNF-α by human PBMCs [206]. Similarly, intranasal administration of *A. fumigatus* AIF (alkali-insoluble cell wall fragments), containing both chitin and β-glucan polysaccharides, as well as chitinase-treated AIF or glucanase-treated AIF in mice, induced lung inflammation and a great influx of immune cells; however, recruitment of neutrophils and eosinophils was only significantly increased in AIF treated mice [207]. Similarly, during in vivo and in vitro experiments with RAW 264.7 macrophages, stimulation with AIF, chitinase-treated AIF or glucanase-treated AIF resulted in increased TNF-α production in the presence of wholesome AIF, suggesting a synergistically interaction of chitin and β-glucan for immune activation [207]. AIF from *A. fumigatus* also had an in vitro inhibitory effect on PBMC cytokine production. Pretreatment of human PBMCs with *A. fumigatus* AIF, and later stimulation with the TLR4 agonist LPS, resulted in decreased secretion of IL-6, when compared to non-pretreated cells [208]. The presence of β-glucan and galactomannan, but not chitin, in AIF, was responsible for the inhibition of IL-6 secretion [208]. These results suggest that the mammalian immune response to *A. fumigatus* is elicited by different cell wall polysaccharides.

Furthermore, the immune response is also modulated by the amount of cell wall polysaccharides, including chitin, present in the cell wall of *A. fumigatus*. Mice that inhaled *A. fumigatus* conidia expressing high levels of chitin (strain Af5517) display reduced gene transcription of adiponectin, an anti-inflammatory mediator [209] in the lungs homogenates compared to mice that aspirated a low chitin expressing strain (Af293) [210]. When adiponectin knockout mice inhaled strain Af5517 conidia, an increase in neutrophil recruitment was observed, and the transcription levels of inflammatory mediators were increased, when compared to strain Af293 [210]. In contrast, WT mice infected with strain Af5517 had an increase in eosinophil recruitment to the lungs, when compared to mice infected with different low chitin-containing strains [211,212] (Figure 5B).

The activation and recruitment of immune cells, primarily eosinophils, mediated by *A. fumigatus* chitin may be γδ T cell-dependent. In the presence of the antifungal drug caspofungin, which induces *A. fumigatus* chitin levels in vitro and in vivo, an increase in lung eosinophil influx was observed in infected mice [211,213]. When γδ T cell-deficient mice were infected with *A. fumigatus* and treated with caspofungin, they displayed increased survival, low fungal burden and low eosinophil counts in the lungs, when compared to nontreated mice [213]. Furthermore, WT mice infected with the high-chitin strain Af5517, presented increased lung eosinophil recruitment when compared to mice infected with the normal chitin strain Af293. In γδ T cell-deficient mice, the eosinophil recruitment in the presence of strain Af5517 was abolished [211]. These studies highlight the importance of γδ T cells in eosinophil recruitment, a response that appears to be dependent on *A. fumigatus* chitin content. Moreover, the increase in eosinophil recruitment to the lungs of mice infected with *A. fumigatus* strain Af5517, is dependent on a Th2 immune response. Mice infected with strain Af5517 exhibited a reduction in CD4 T cells, which produce IFN-γ, and a transcriptional increase in Th2 chemokines and cytokines (CCL11 and CCL22) in the lungs, when compared to infection with strain Af293 [212].

Although the high-chitin strain Af5517 elicited an increased early inflammatory response in an infection model of aspirated conidia [210,211], the development and progression of disease in a mouse model of invasive aspergillosis (IA), is less detrimental than for strain Af293, as assessed by mouse survival, disease score and fungal burden in the murine lung [214]. Such difference may be related to a delay in the growth of strain Af5517 [214], or the increased recognition and clearance by immune cells; indeed higher concentrations/differential exposure of chitin and β-glucans trigger recognition by Dectin-1 and phagocytosis [215,216]. Alternatively, in order to promote IA, mice had to be immunosuppressed, so the lack of neutrophils in this murine model may also be related to the observed disease outcome when infected with both high- and low-chitin *Aspergillus* isolates. O’Dea et al. (2014) showed that the observed reduction in disease severity, in a model of IA infection, was related to the presence of eosinophils. BALB/c mice that aspirated Af5517 conidia, but not Af293, displayed an increase in eosinophil numbers in BALF. Additionally, eosinophil-deficient mice that were repeatedly challenged with *A. fumigatus* strain Af5517, showed increased survival, low disease score and decreased lung fungal burden when compared to neutropenic mice infected with the same strain [212] (Figure 5B).

These studies suggest that chitin present in the *A. fumigatus* cell wall induces the recruitment of eosinophils, which are the main cells related to allergic inflammatory responses. Intraperitoneal immunization of C57BL/6 mice with *A. fumigatus* allergen extract (1WCF) with crab shell chitin as adjuvant, led to an increase in total IgE and specific serum IgE and IgG1 [217]. These results indicate an allergic property of 1WCF that is enhanced in the presence of chitin. In agreement, the activity of eosinophil peroxidase (EPO) in BALF cell pellet lysate was increased during 1WCF immunization with chitin as adjuvant, supporting an increase in the number of eosinophils in the lung [217]. Strong et al. (2002) employed a different approach to unveil the allergic properties of chitin during *Aspergillus* infection. Mice were immunized with *A. fumigatus* allergen extract (Afu), using alum as an adjuvant, and then intranasally challenged with Afu and purified chitin (Sigma-Aldrich) or Afu and PBS. A reduction in total serum IgE, specifically in Afu IgG, as well as in peripheral blood eosinophils, was observed in mice challenged with Afu and chitin when compared to mice challenged with Afu and PBS. Furthermore, the levels of Th1 cytokines (IL-12, TNF-α and IFN-γ) were significantly increased in the spleen of mice challenged with Afu and chitin, while IL-4 levels were decreased when compared to mice challenged with Afu and PBS [218]. In agreement, the respiratory capacity and lung histology of mice challenged with Afu and chitin were preserved when compared to those of mice challenged with Afu and PBS [218]. These results suggest a modulation of the allergic response elicited by *A. fumigatus* extracts that is somewhat dependent on the phase of chitin administration.

Differences in chitin source and size, as well as the experimental design could explain the differences observed in the immune modulation exerted by chitin in both studies that used only immunized mice and the same adjuvant. Similar to Dubey et al. (2015), Roy et al. (2013) showed that mice sensitized with a combination of *A. fumigatus* antigens and chitin purified from crab shells presented a strong allergic response. They observed an increase in eosinophilia in the lungs as well as expression of Th2 cytokines (IL-4, IL-5 and IL13), and increased serum IgE levels when compared to mice treated with either chitin or *A. fumigatus* antigens alone [219]. The immunological response in mice sensitized with *A. fumigatus* antigens and crab shell chitin was dependent on chitin complement opsonization and cleavage of C3, leading to the activation of the C3a receptor (C3aR). A similar opsonization-dependent response to *A. fumigatus* purified chitin was also observed in vitro in PBMC-treated cells [206]. Mice sensitized with *A. fumigatus* antigens and crab shell chitin also showed a less tolerogenic phenotype in lung plasmacytoid dendritic cells (pDCs) that were related to an increased inflammatory T cell response in the lungs, and which consisted of CD4^+^ IL-4^+^ and CD4^+^ IL-17^+^ T cells. This increased inflammatory response was associated with the presence of C3 and C3aR activation, as the corresponding C3KO mice presented a more tolerogenic response to *A. fumigatus* antigens and crab shell chitin, which was accompanied by an increase in CD4^+^ Foxp3^+^ T cells, and a reduction in CD4^+^ IL-4^+^ and CD4^+^ IL-17^+^ T cells [219].

Furthermore, the use of *A. fumigatus* CS deletion mutants highlighted the importance of chitin and cell wall organization for fungal interaction with the mammalian immune system. de Jesus Carrion and colleagues (2019) showed that the *A. fumigatus* Δ*AfchsA* Δ*AfchsC* Δ*AfchsB* Δ*AfchsG* (division 1) and Δ*AfcsmA* Δ*AfcsmB* Δ*AfchsD* Δ*AfchsF* (division 2) strains were more susceptible to human neutrophil killing, as assessed by the XTT viability of fungal cells, when compared to the parental strain. In a murine model of corneal infection, *A. fumigatus* strains deleted for division 1 or 2 of CS-encoding genes, caused a reduction in virulence as measured by a decrease in CFU in the eyes of mice and less corneal disease when compared to the parental strain [98]. Interestingly, neutrophil-mediated killing of fungal hyphae depended on the activity of acidic mammalian chitinase (AMCase), an enzyme that hydrolyzes chitin. Indeed, the fungicidal properties of neutrophils in the presence of chitinase inhibitors or when using neutrophils from AMCase^−/−^ mice were impaired [98]. A disturbance in chitin synthesis due to the absence of CS-encoding genes causes restructuring/reorganization of the fungal cell wall. Indeed, the deletion of *AfcsmA, AfcsmB* or both of these genes resulted in the disruption or complete loss of the *A. fumigatus* conidial rodlet layer, which in turn exposed mannan and chitin, resulting in an increased immunological response [108]. Indeed, DCs cultivated with the single and double deletion strains of these CS-encoding genes had increased surface activation and displayed an increase in maturation markers, such as CD80, CD86, CD40, CD83 and in the expression of the major histocompatibility complex class II (HLA-DR) [108]. The *A. fumigatus* CS encoded by gene *AfchsG* from division 1 seems to play a major role in chitin biosynthesis in conidia, as deletion of the corresponding gene caused a 60% reduction in conidial chitin, whereas a small reduction in mycelial chitin levels were observed [103]. Strains Δ*AfchsB* and Δ*AfchsC*, also deleted for division 1 CS-encoding genes, display a subtle reduction in mycelial chitin, whereas the Δ*AfchsA*, Δ*AfchsB* and Δ*AfchsC* strains showed a small reduction in conidial chitin [103]. A strain deleted for all four CS-encoding genes from division 1 (Δ*AfchsA* Δ*AfchsC* Δ*AfchsB* Δ*AfchsG*) was as virulent (assessed through survival curves and histological analyses) as the parental strain in both immunosuppressed murine and insect (*Galleria mellonella*) models of infection [103]. This can be explained by the insignificant reduction in mycelial and conidial chitin content in the quadruple mutant when compared to the WT strain. Deletion of the *A. fumigatus* CS-encoding gene Δ*AfchsF* from division 2 resulted in a strain with a 25% reduction in mycelial chitin, whereas strains Δ*AfchsF*, Δ*AfcsmB* and Δ*AfcsmA* (deleted for division 2 CS-encoding genes) displayed a reduction in cell wall chitin content in the conidia, with Δ*AfcsmA* mutant showing the greatest reduction (~80%) when compared to the parental strain. The quadruple mutant of division 2 CS-encoding genes (Δ*AfcsmA* Δ*AfcsmB* Δ*AfchsF* Δ*AfchsD*) showed comparable chitin content (in mycelia and conidia) than the parental strain, although chitin microfibrils showed significant structural differences, which could account for the observed hypovirulent phenotype of this strain in both immunosuppressed murine and insect models of aspergillosis. The structural differences were not observed for the class 1 quadruple deletion strain, suggesting that CS from division 2 is more important for chitin structure and cell wall integrity than CS from division 1 [103].

In addition to gene-encoding proteins involved in chitin metabolism, *A. fumigatus* gene encoding proteins involved in cell wall morphogenesis and biosynthesis also affect cell wall chitin content and interaction with mammalian immune cells. The *A. fumigatus Afcps1* gene was identified from a genome-wide random insertional mutagenesis screening as an important regulator of morphogenesis [220]. *Afcps1* encodes for capsular polysaccharide synthase 1 (Cps1), which is required for normal colony development. The absence of *Afcps1* resulted in reduced α-glucan, β-glucan, and chitin levels in *A. fumigatus* mycelia, as well as in a decreased transcription of CS-encoding genes [220]. Incubation of *ΔAfcps1* conidia with BMDMs revealed that the absence of *Afcps1* resulted in greatly induced BMDM cellular activation and upregulation of genes involved in killing, biological adhesion and the production of cytokines and chemokines (*IL1b*, *IL12b*, *IL10* and *Ccl22,* among others) [220]. Even though the *ΔAfcps1* strain presented reduced expression of cell wall α-glucan, β-glucan and chitin, the immune stimulatory properties of this strain were significantly enhanced when compared to the WT strain. Similarly, deletion of *ssdA*, encoding a multifunctional RNA-binding protein involved in conidial and mycelial trehalose metabolism in *A. fumigatus*, resulted in a strain with increased trehalose, decreased chitin and β-1,3-glucan levels, while *ssdA* overexpression (OE::*ssdA*) caused decreased trehalose, increased chitin and reduced β-1,3-glucan levels [135]. In a murine model of invasive aspergillosis, the OE::*ssdA* (high chitin content) strain had decreased virulence, while the *Δssda* (low chitin content) strain was as virulent as the WT strain. Increased mice survival, reduction in immune infiltrate in BALF and a reduction in lung fungal growth were observed for mice infected with the OE::*ssdA* strain [135]. Furthermore, the absence of the regulatory subunit TslA (*ΔtslA*) involved in trehalose biosynthesis in *A. fumigatus*, resulted in a strain with decreased trehalose and β-glucans levels, and increased chitin levels [21]. Indeed, the *ΔtslA* strain presented increased chitin synthase activity, due to negative regulation in the CS *AfcsmA* (Afu2g13440) by TslA [134]. In a murine model of invasive pulmonary aspergillosis, the *A. fumigatus ΔtslA* strain was as virulent as the WT strain. However, mice infected with the *ΔtslA* strain displayed an increased inflammatory state, with increased inflammatory lesions in the lungs, greater recruitment of neutrophils and macrophages to BALF, and increased secretion of inflammatory cytokines and chemokines (TNF-α, MIP-1α and CXCL1) [134]. This result contradicts a study that was carried out by the same group in 2019, where the authors observed that overexpression of SsdA resulted in increased fungal cell wall chitin and a reduction in virulence [135]. Similarly, the *ΔzipD* strain, which codes for a transcription factor involved in the CPE and the calcium-calcineurin pathway, also presented decreased β-1,3-glucan content, increased levels of chitin and a thicker cell wall in the presence of caspofungin when compared to the WT strain, suggesting that ZipD is important for cell wall maintenance and integrity. In a murine model of invasive pulmonary aspergillosis, the ∆*zipD* strain was avirulent, with conidia from this stain being more susceptible to BMDM-killing, which was accompanied a by higher production of the proinflammatory cytokines IL-12p40, Il-6, IL-1β and TNF-α. A similar inflammatory phenotype was observed in immunocompetent mice, where infection with ∆*zipD* caused a greater influx of innate and adaptive inflammatory cells to the lungs, accompanied by an increased secretion of proinflammatory cytokines and a decrease in fungal burden. The authors of this study suggested that ZipD-dependent cell wall organization is important for *A. fumigatus* immune evasion, although additional mechanisms that are regulated by this transcription factor cannot be excluded [193]. Together, these studies suggest that the deletion and overexpression of different genes, causes additional alterations in fungal cell development and fitness, which can impact cell wall integrity and composition, and in turn promote different immune responses. It is also worth noting that the aforementioned studies show that a reduction in chitin did not affect virulence, whereas an increase in cell wall chitin caused a reduction in virulence. It would be interesting to measure cell wall thickness in all these deletion strains and determine whether a correlation with strain-specific virulence exists. Alternatively, each of the aforementioned gene deletions caused effects other than cell wall remodeling on the fungal cell, which could also account for the observed differences in virulence. For example, the Δ*Afcps1 A. fumigatus* strain showed compact and wrinkled colony shapes, reduced colony size, conidiation and delayed fungal growth when compared to the parental strain [220]. The *ΔssdA* and OE::*ssdA* strains also displayed a decrease in mycelial radial growth and reduced biomass in liquid culture when compared to the parental strain [135]. Furthermore, *ΔssdA* conidia germinate faster, and OE::*ssdA* conidia germinate slower when compared to the parental strain. The *ΔtslA* displayed delayed conidia germination when compared to the parental strain [134]. Together, the observed differences in immune responses to the deletion strains may be a combination of defects, including cell wall organization, growth and development. 

## 6. CS Gene Manipulation and Biotechnological Applications

Several species from the *Aspergillus* genus have important biotechnological and biomedical applications, due to their high metabolic diversity, and subsequent production and secretion of industrially relevant enzymes and secondary metabolites [221]. *Aspergillus* species, such as *A. niger, A. oryzae* and *A. terreus* are routinely used for citric acid production, oriental food production (such as soy sauce, sake brewing and soybean paste), and the production of statins, which are cholesterol-lowering drugs [222,223].

With the acquisition of more advanced genetic tools, the analysis of fungal transcriptional responses during industrial processes can be assessed. Yin and colleagues, 2016, demonstrated that during citric acid production, *A. niger* had an increase in the expression of cell wall related genes, including the CS-encoding gene *AngchsF* (An12g10380) [101]. This gene was also significantly expressed during glucoamylase synthesis [224], suggesting that chitin synthesis, perhaps not surprisingly, is also important for fungal morphology under industrial culture conditions.

Indeed, genetic engineering targeting CS-encoding genes, and the productivity of the resulting strains under industrial culture conditions were carried out. In *A. niger*, RNA interference (RNAi) was used to silence *AngchsC* (An8g04350) expression, resulting in a strain with decreased conidia production and chitin concentrations, highly compact mycelial pellets in submerged cultures and increased production of citric acid [73]. Needless to say, high citric acid-producing strains in bioreactors are highly desired for industrial citric acid production.

In *A. oryzae*, Müller and colleagues showed that the deletion of a CS-encoding gene can have positive effects on the rheological features of the culture [225,226]. In submerged cultures, *AochsB* (∆AO090701000589) strain presented decreased hyphal growth, when compared to A1560 WT strain, and no differences on α-amylase yield were observed between the strain [226]. The *AochsB* deletion strain has more hyperbranched hyphae [72,225,226] and a ~88% reduction in extension rate when compared to the WT strain, thus, having severe defects in hyphal extension [225]. The fermentation broth of the *AochsB* deletion strain was significantly less viscous when compared to the one from the WT strain, a feature that is desired for improving submerged culture conditions and increasing enzyme yield [226].

The aforementioned studies further emphasize the close relationship between chitin synthesis and fungal morphology. Unfortunately, not many studies have investigated the role of cell wall polysaccharide biosynthesis in optimizing bioreactor and culture conditions, although the aforementioned studies clearly show some promising results. Targeting cell wall chitin and other sugar biosynthesis is therefore, an interesting strategy for improving yields of fungal products as well as improving culture conditions, especially those that are dependent on fungal morphology. 

## 7. Summary and Conclusions

Chitin is a structural component of the fungal cell wall, which, together with other polysaccharides, such as glucan, confers cell rigidity and shape, is essential for fungal survival and interaction with the extracellular space. Chitin biosynthesis is catalyzed by different classes of chitin synthases (CS) in *Aspergillus* species, and the expression of these enzymes is dependent on the stage of fungal growth and the presence of extracellular cues, such as cell wall stressors. Up to eight CS-encoding genes can be present in the genomes of *Aspergillus* species. CS are crucial for fungal growth and development, as deletion of the respective genes often results in strains with morphological defects in hyphal structure and growth as well as conidia formation. Deletion of some CS-encoding genes did not result in an obvious phenotype, and their specific function remains unknown. Figure 3 shows an overview of CS genes and their functions in *Aspergillus* species. 

Although the metabolic steps of chitin biosynthesis are well described, processes involved in the initiation of chitin biosynthesis remain to be elucidated in all *Aspergillus* species (Figure 1). Similarly, how CS are localized to the extending hyphal tip or to hyphal branches as well as to conidiophores remains to be investigated in many *Aspergillus* species. The exception is *A. nidulans*, where the transport of some CS to the actively growing hyphal tip was shown to be dependent on kinesins and occurred along microtubules and actin filaments. Nevertheless, further investigation is required to determine the localization and transport of cell wall modifying enzymes to sites of growth and or conidiation in *Aspergillus* species.

Chitin biosynthesis is regulated by many different pathways, which ensure cell wall integrity, respond to extracellular stresses and provide the energy to support fungal growth and development (Figure 4). Crosstalk exists between these pathways, which accommodate different signals and allow the cell to adequately respond to these cues while continuing growth.

Considering the importance of chitin biosynthesis for fungal development and the complexity of regulation underlying chitin biosynthesis, it is perhaps not a surprise that these processes are intrinsically linked to antifungal drug resistance and interaction with mammalian immune cells. Indeed, many antifungal drugs or a combination thereof, target the synthesis of cell wall polysaccharides (CWP), including chitin, in order to disrupt fungal growth and cause cell death. CWP are interesting targets for the development of antifungal strategies, as these sugars are not present in mammalian cells. Unfortunately, inhibiting the synthesis of one CWP can lead to an increase in another CWP, thus, conferring fungal resistance to the drug. The most prominent example of this is the CPE, where inhibition of glucan biosynthesis leads to an increase in CW chitin levels and improved growth in the presence of the echinocandin caspofungin. The mechanism underlying the CPE has proven itself to be extremely complex, and recent studies have shown that many regulators are involved in sustaining the CPE in the presence of high concentrations of caspofungin. Our understanding of the CPE is far from complete, and additional studies are required to fully understand the fungal cellular mechanisms that support the CPE in order to counteract this phenomenon and maximize the potential of echinocandins in treating invasive fungal diseases.

Similarly, chitin, in conjunction with other CWP, is an important target that is recognized by mammalian immune cells and triggers an adaptive response. Deletion of genes coding for CS or regulators of chitin biosynthesis often results in strains with a significant reduction in virulence, although this may be due to the strain-specific morphological defects and a general reduction in fitness caused by the respective deletion. Nevertheless, chitin and other CWP are crucial for triggering an immune response and keeping opportunistic fungal infections at bay, making it a possible therapeutic target for antifungal strategies, such as vaccines, although this has not been successful to date.

Manipulation of chitin biosynthesis is not only relevant for biomedical applications, but a few studies have shown a potential for biotechnological applications, where genetic interference of chitin biosynthesis resulted in increased secretion of a valuable product or improved culture conditions. Unfortunately, not many studies have addressed this, and manipulation of fungal cell wall properties certainly makes an interesting case for strain engineering in biotechnological applications.

Together, the mechanisms underlying chitin biosynthesis are crucial for fungal homeostasis and, thus, for antifungal drug resistance, interaction with mammalian immune responses and biotechnological applications. Fungal chitin metabolism, therefore, continues to be a highly interesting topic that is worth investigating even after more than 30 years of research in this area.

## Figures and Tables

**Table 1:** Chitin Synthase genes on *Aspergillus* species classified into divisions and classes.

*Aspergillus* Specie	Chitin Synthase Gene	ID Gene ^#^	Alias	Division	Class
*A. nidulans*	*chsA*	AN7032	-	1	II [68]
	*chsB*	AN2523	-	1	III [68]
	*chsC*	AN4566	-	1	I [69]
	*chsD*	AN1555	*chsE*	2	IV [43,92]
	*chsF*	AN4367	-	1	III [93]
	*chsG*	AN1046	-	2	VI [79]
	*csmA*	AN6318	*chsD*	2	V [94]
	*csmB*	AN6317	-	2	VII [95]
*A. fumigatus*	*chsA*	Afu2g01870	-	1	I [70]
	*chsB*	Afu4g04180	-	1	II [70]
	*chsC*	Afu5g00760	-	1	III [70]
	*chsD*	Afu1g12600	-	2	VI [96]
	*chsF*	Afu8g05630	-	2	IV [70,93]
	*chsG*	Afu3g14420	-	1	III [97]
	*csmA*	Afu2g13440	*chsE*	2	V [71,98,99]
	*csmB*	Afu2g13430	-	2	VII [71]
*A. oryzae*	*chsA*	AO090012000084	-	1	III [100]
	*chsB*	AO090701000589	-	1	III [72]
	*chsC*	AO090011000449	-	1	I [72]
	*chsY*	AO090026000323	-	2	V [100]
	*chsZ*	AO090026000321	-	2	VII [100]
*A. niger*	*chsC*	An8g04350	-	1	III [73]
	*chsF*	An12g10380	-	1	III [101,102]

^#^ The gene IDs were extracted from the database FungiDB and were adopted during the review as CS gene designations.

## Data Availability

Not applicable.
